# Affinity Proteomics and Deglycoproteomics Uncover Novel EDEM2 Endogenous Substrates and an Integrative ERAD Network

**DOI:** 10.1016/j.mcpro.2021.100125

**Published:** 2021-07-29

**Authors:** Cristian V.A. Munteanu, Gabriela N. Chirițoiu, Marioara Chirițoiu, Simona Ghenea, Andrei-Jose Petrescu, Ştefana M. Petrescu

**Affiliations:** 1 Department of Bioinformatics and Structural Biochemistry, Institute of Biochemistry, Bucharest, Romania; 2 Department of Molecular Cell Biology, Institute of Biochemistry, Bucharest, Romania

**Keywords:** EDEM2, proteomics, pulsed-SILAC, glycoproteomics, MS, melanoma, ERAD, ER quality control, ACN, acetonitrile, AE, affinity enrichment, CANX, calnexin, CCDC47, coiled-coil domain–containing protein 47, cDNA, complementary DNA, DERL1, Derlin-1, DMEM, Dulbecco's modified Eagle's medium, DNAJB12, DnaJ homolog subfamily B member 12, E2D, plasmid encoding the C terminus missing EDEM2, EDEM2, ER degradation–enhancing α-mannosidase-like protein 2, EndoH, endo-β-*N*-acetylglucosaminidase H, ER, endoplasmic reticulum, ERAD, ER-associated degradation, ERLEC1, ER lectin 1, ERQC, endoplasmic reticulum quality-control, FA, formic acid, FDR, false discovery rate, GluC, glucosidase C, GO, Gene Ontology, gpERAD, glycoprotein ERAD, HA, hemagglutinin, HCD, higher collisional dissociation, HLA, human leukocyte antigen, ITGA1, integrin alpha-1, LBB, lectin-binding buffer, LFQ, label-free quantification, MAN1B1, mannosyl-oligosaccharide 1,2-alpha-mannosidase, *N*-glycoFASP, *N*-glyco filter–aided sample preparation, NSC, normalized spectral count, ON, overnight, PCDH2, protocadherin 2, pSILAC, pulse stable isotope labeling with amino acids in cell culture, PSM, peptide spectrum match, RES, relative evolution score, RT, room temperature, SAINT, significance analysis of interactome, SEL1L, protein sel-1 homolog 1, siEDEM2, siRNA sequence targeting EDEM2, siScr, noncoding RNA sequence, scramble, TMX, thioredoxin transmembrane member, TRP-1, melanoma antigen gp75, TXNDC11, thioredoxin domain–containing protein 11, UGGT, uridine diphosphate–glucose:glycoprotein glucosyltransferase, WB, Western blot

## Abstract

Various pathologies result from disruptions to or stress of endoplasmic reticulum (ER) homeostasis, such as Parkinson's disease and most neurodegenerative illnesses, diabetes, pulmonary fibrosis, viral infections, and cancers. A critical process in maintaining ER homeostasis is the selection of misfolded proteins by the ER quality-control system for destruction *via* ER-associated degradation (ERAD). One key protein proposed to act during the first steps of misfolded glycoprotein degradation is the ER degradation–enhancing α-mannosidase-like protein 2 (EDEM2). Therefore, characterization of the EDEM2-associated proteome is of great interest. We took advantage of using melanoma cells overexpressing EDEM2 as a cancer model system, to start documenting at the deglycoproteome level (N-glycosites identification) the emerging link between ER homeostasis and cancer progression. The dataset created for identifying the EDEM2 glyco clients carrying high mannose/hybrid N-glycans provides a comprehensive N-glycosite analysis mapping over 1000 N-glycosites on more than 600 melanoma glycoproteins. To identify EDEM2-associated proteins, we used affinity proteomics and proteome-wide analysis of sucrose density fractionation in an integrative workflow. Using intensity and spectral count–based quantification, we identify seven new EDEM2 partners, all of which are involved in ER quality-control system and ERAD. Moreover, we defined novel endogenous candidates for EDEM2-dependent ERAD by combining deglycoproteomics, stable isotope labeling with amino acids in cell culture–based proteomics, and biochemical methods. These included tumor antigens and several ER-transiting endogenous melanoma proteins, including integrin alpha-1 and protocadherin 2, the expression of which was negatively correlated with that of EDEM2. Tumor antigens are key in the antigen presentation process, whereas integrin alpha-1 and protocadherin 2 are involved in melanoma metastasis and invasion. EDEM2 could therefore have a regulatory role in melanoma through the modulation of degradation and trafficking in these glycoproteins. The data presented herein suggest that EDEM2 is involved in ER homeostasis to a greater extent than previously suggested.

## Highlights

• EDEM2 affinity proteomics and proteome-wide analysis of sucrose density fractionation.
• New EDEM2 partners involved in ER quality control and ERAD.
• Soluble tyrosinase degradation is EDEM2 dependent in melanoma cells.
• Glycoproteomics and SILAC-based proteomics reveal novel EDEM2 potential substrates.

## Graphical Abstract

**Figure:**
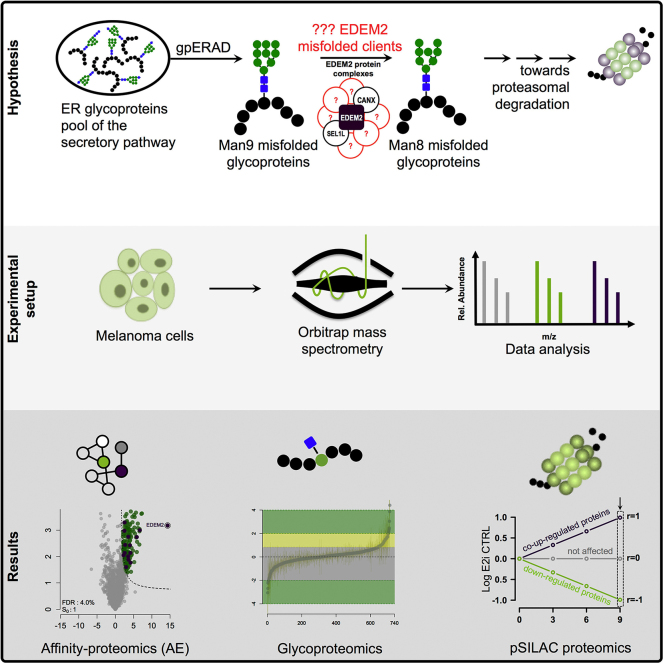

## In Brief

Various pathologies including neurodegenerative diseases and cancers result from disruptions to or stress of ER homeostasis. One key protein proposed to act in quality control processes maintaining ER homeostasis is EDEM2. Using affinity proteomics and sucrose-density sedimentation, we identified several new EDEM2 partners involved in quality control. Moreover, we defined novel endogenous candidates for EDEM2-dependent degradation by combining glycoproteomics and SILAC-based proteomics. Our data suggest that EDEM2 is involved in ER homeostasis to a greater extent than previously thought.

The endoplasmic reticulum (ER) quality-control (ERQC) system and ER-associated degradation (ERAD) machinery work as interconnected networks to prevent the accumulation of unfolded or misfolded polypeptides and maintain the ER proteostasis. These networks include multiple factors controlling molecular events such as burying hydrophobic patches of amino acids in the protein core, formation of the disulfide bridges, N-glycan binding, and trimming of glycoproteins for extraction and degradation of misfolded proteins (1, 2). Newly produced polypeptides can undergo chaperone-assisted folding up to several binding-release rounds (2); however, unproductive folding of the protein leads to its rerouting toward ERAD and degradation by the ubiquitin–proteasome pathway (3, 4). The selection and degradation steps for misfolded glycoproteins involve N-glycan processing by ER alpha-glucosidases I and II and members of the glycosylhydrolase family 47 sharing an (αα)7-barrel catalytic domain of class I mannosidases (5). Key proteins were previously proposed to act in the selection and degradation of misfolded glycoproteins. Thus, uridine diphosphate–glucose:glycoprotein glucosyltransferase (UGGT) recognizes folding defects through its sensor region (6) and reglucosylates the incompletely folded glycoprotein, diverting the substrate toward another attempt of lectin-based chaperone–assisted folding process. Members of the glycosylhydrolase family 47 family, ER degradation–enhancing mannosidase-like proteins (EDEMs) 1, 2, and 3 were shown to be important players in ERAD (7, 8). In particular, EDEM2 has been proposed as the initiator of the glycoprotein ERAD (gpERAD) for misfolded glycoproteins (9).

Since its initial identification, a number of reports showed evidence that EDEM2 is involved in the selection and degradation of misfolded glycoproteins: the accelerated degradation of canonical misfolded glycoproteins in overexpression experiments (10, 11), the specific association with calnexin (CANX), thioredoxin domain–containing protein 11 (TXNDC11) (12), the interaction with several conventional ERAD substrates (10, 11), and the rather controversial association with protein sel-1 homolog 1 (SEL1L) (9, 13, 14). Although initial investigations indicated that EDEM2 does not possess mannosidase activity and cannot recognize nonglycosylated versions of canonical ERAD substrates, subsequent reports challenged these aspects, suggesting that this protein is involved in gpERAD by trimming Man9 to Man8 glycoforms (9, 12, 13, 15). Altogether these point to a more complex landscape in EDEM2 partner recognition and selection. Moreover, to date, no endogenous candidate substrates were proposed for EDEM2. This would be of particular interest in melanoma cells, since ERAD and the ubiquitin–proteasome pathway can generate peptides amenable to human leukocyte antigen (HLA) presentation, thus contributing to the immunopeptide repertoire specific to cancer cells (16).

Using interaction proteomics, here, we have characterized the EDEM2-associated proteins in melanoma cells and defined novel potential endogenous EDEM2 candidate substrates in a system-wide functional proteomics workflow and found that the degradation of a glycosylated misfolded tyrosinase mutant observed in some melanomas and oculocutaneous albinism patients (17, 18, 19, 20, 21, 22, 23) is EDEM2 dependent. The experiments also reveal novel potential EDEM2 endogenous substrates and characterize one of the largest endo-β-*N*-acetylglucosaminidase H (EndoH)–sensitive *N*-deglycoproteome from melanoma cells. Moreover, some of the candidates were also validated using pulse stable isotope labeling with amino acids in cell culture (pSILAC) proteomics, Western blot (WB), and confocal microscopy.

## Experimental Procedures

### Plasmid Construction, Stable Cell Lines Generation, and Expansion

The plasmid encoding the C terminus missing EDEM2 (E2D) was obtained by cloning the corresponding complementary DNA (cDNA) into XhoI–NotI sites of pLPCX with specific designed primers, encoding also for hemagglutinin (HA) tag.

The cDNA corresponding to the human EDEM2 with HA tag, for inducible cell line generation, was cloned in XhoI–SalI/NotI sites of *pENTR4-FLAG* (w210–2) (plasmid #17423; Addgene), from which the FLAG tag was removed. EDEM2-pENTR4 was recombined with the destination vector *pLenti CMV/TO Puro DEST* (670-1) (plasmid #17293; Addgene), using the Invitrogen Gateway LR Clonase II enzyme mix. The recombination product was transformed into *Stbl3* competent cells for clonal selection of EDEM2-pLenti CMV to PURO.

A375 melanoma cells (European Collection of Animal Cell Cultures) were further used to obtain the following cell lines.

A375 soluble tyrosinase EDEM2-inducible melanoma cell line (further denoted as A375-ST-TYR-E2i) was generated using the BLOCK-iT Inducible H1 Lentiviral RNAi System (K4925-00; Thermo Fisher Scientific), according to the manufacturer's protocol. We generated first, A375-ST-TYR-TetR cell line, by transducing A375-ST-TYR (24) with pLenti 6/TetR lentiviral particles, for 24 h, followed by selection with 5 μg/ml blasticidin (Invivogen), for 2 to 3 passages. Cells were routinely grown in 2.5 μg/ml blasticidin until single-cell cloning. Three different clones were selected using a FACS ARIA (BD Bioscience) sorter for further transduction with EDEM2 lentiviral particles. For this, A375-ST-TYR-TetR clones seeded at 30 to 40% confluence were transduced with EDEM2-pLenti CMV/TO Puro lentiviral particles, in the presence of 6 to 8 μg/ml polybrene (Sigma), for 24 h. Cells were selected using 6 μg/ml puromycin (Invivogen) for 2 to 3 passages and routinely maintained in 2 μg/ml puromycin.

The A375-pLPCX (A375-C), A375 HA-tagged EDEM2-pLPCX (A375-E2), A375-pLNCX2 (A375-CTRL), A375 WT tyrosinase (A375-WT-TYR), A375 soluble tyrosinase (A375-ST-TYR), and A375 deglycosylated tyrosinase (A375-Δall-TYR) stable cell lines were obtained using a retroviral amphotropic system as previously described (24, 25). These cells were cultivated in Dulbecco's modified Eagle's medium (DMEM) (Gibco) supplemented with 10% fetal bovine serum (Gibco) and selection antibiotics, geneticin, or puromycin.

### pSILAC Cell Lines

For pSILAC experiments, A375-ST-TYR-E2i cells were grown in arginine- (R)/lysine- (K) free DMEM (Gibco) media supplemented with either medium R:^13^C6-l-arginine (R6) or heavy R:^13^C6^15^N4-l-arginine (R10), and light K (K0) and proline. These were grown until complete labeling (verified by MS). The cells were split in individual dishes corresponding to each chase time, and at the start of the experiment, the media were changed with DMEM supplemented with medium K:^2^H4-l-lysine (K4) or heavy K:^13^C6^15^N2-l-lysine (K8) and light R (R0). In one of the replicates, tetracycline was added (1 μg/ml) to induce EDEM2 expression. Cells were harvested at the indicated chase time points and mixed. Two replicates with label swap were performed.

### Transient Transfection of siRNA and Plasmids

For knockdown experiments, A375-ST-TYR melanoma cells were reverse transfected with a mix off three siRNA sequences targeting EDEM2 (siEDEM2) (sc-77226; Santa Cruz Biotechnology) or a noncoding RNA sequence, scramble (siScr) (sc-37007; Santa Cruz Biotechnology) using Lipofectamine RNAiMAX (Invitrogen) for 72 h.

For overexpression experiments, A375 melanoma cells stably expressing tyrosinases WT-TYR, ST-TYR, Δall-TYR, or pLNCX2 (CTRL) were transfected with plasmids encoding for full-length EDEM2 (E2), E2D, or pLPCX (C).

### Treatment by Inhibitors

A375-ST-TYR-E2i were treated with 20 μM MG132 (sc-201270; Santa Cruz Biotechnology) for 6 h at 37 °C or overnight (ON) with 30 μM kifunensine (sc-201364; Santa Cruz Biotechnology).

### Cycloheximide-chase Experiments

For cycloheximide chase, A375-ST-TYR-E2i treated or not with tetracycline and A375-ST-TYR transfected with siEDEM2 or siScr were grown in DMEM supplemented with 50 μM cycloheximide for the indicated time points. Harvested cells were lysed in 1% Triton X-100 buffer (50 mM Hepes, 1.5 mM MgCl_2_, 50 mM NaCl, 1 mM EDTA, 1% Triton X-100, and pH 7.4), supplemented with protease inhibitors (Roche) and equal amounts of total protein were separated by SDS-PAGE, transferred on nitrocellulose membranes, and probed with the specific antibodies.

### Affinity Enrichment of EDEM2 or its Associated Proteins

A375-E2 or A375-C cells were lysed in 1% digitonin lysis buffer (1% digitonin [w/v], 50 mM Tris–HCl, pH 7.4, 150 mM NaCl, and 5 mM EDTA), as previously described (25). Samples were cleared, and the supernatant was incubated with an anti-HA mouse monoclonal antibody (clone 16B12) ON at 4 °C or with specific antibodies for associated proteins: CANX (ab22595), PDIA4 (sc-135901), ER lectin 1 (ERLEC1) (ab181166), SEl1L (sc-48081), and Derlin-1 (DERL1) (D4443). Protein complexes were isolated on protein G or A Sepharose beads for 2 h at 4 °C and further washed three times with wash buffer (0.1% digitonin [w/v], 50 mM Tris–HCl, pH 7.4, 150 mM NaCl, and 5 mM EDTA). Proteins were eluted twice by incubating the Sepharose beads with soft elution buffer (50 mM Tris–HCl, pH 8, 0.2% SDS, and 0.1% Tween-20) (26) at room temperature (RT) for 15 min in a 1:2 (v:v) ratio and respective 1:1 (v:v) ratio of beads to soft elution buffer. Proteins from the combined eluted fractions were further separated by SDS-PAGE and subjected to trypsin in-gel digestion. For WB, the beads were boiled for 5 min in 1× Laemmli buffer, 1:2 (v:v) ratio beads:buffer, separated in polyacrylamide gels, transferred on nitrocellulose membranes, and probed with specific antibodies.

### In-gel Trypsin Digestion

Protein in-gel digestion was performed as previously described (24, 27). Briefly, gel slices were washed with 50% acetonitrile (ACN), reduced with 10 mM DTT at 56 °C for 45 min, and alkylated with 55 mM iodoacetamide at RT for 45 min. Gel slices were dehydrated, and trypsin was digested ON at 37 °C. The digestion was quenched with 5% formic acid (FA) in 50% ACN (1:2, v:v) and peptides were extracted by incubating once in the same buffer and then in 5% FA in ACN. The combined fractions were vacuum concentrated and kept at −20 °C until further analysis.

### EndoH Deglycoproteome Isolation

A375-ST-TYR melanoma cells transfected with siEDEM2 or a noncoding RNA sequence (siScr) were subject to *N*-glyco filter–aided sample preparation (*N*-glycoFASP) enrichment protocol as previously described (28), with slight modifications. Briefly, cells were lysed with extraction buffer (500 mM Tris–HCl, pH 8.50, 6 M guanidine hydrochloride, 5 mM Tris(2-carboxyethyl)phosphine, and 10 mM chloroacetamide), and proteins from each sample were captured on a 10 K centricon unit. The filters were washed three times with digestion buffer (50 mM ammonium bicarbonate) and then subject to protease ON digestion, either with glucosidase C (GluC) (1:15, w:w) at 25 °C, or with trypsin (1:50, w:w), protease:protein ratio at 37 °C. The resulting peptides were collected in lectin-binding buffer (LBB) containing 20 mM Tris–HCl, pH 7.50, 500 mM NaCl, 1 mM MnCl_2_, and 1 mM CaCl_2_ and transferred to new 30 K centricon filters and incubated at RT for 60 min with a solution of 2× LBB concanavalin A in a ratio of 1.0:2.1 (w:w) peptide:lectin. Subsequently, the unbound peptides were removed by centrifugation at 10,000*g*, and the filters were washed four times with 0.2 ml LBB. Deglycosylation was performed by ON incubation of the filters at 37 °C, with 65 μl of 2 U/μl EndoH (1:2, peptides:enzyme) in G3 buffer (50 mM sodium acetate, pH 6.00). The resulting glycopeptides were recovered by centrifugation at 10,000*g* for 20 min and by two additional sequential washes with G3 buffer. Fractions were combined and further subjected to C18 StageTips desalting as described further.

### pSILAC Sample Preparation for nanoLC–MS/MS Analysis

At each time point, an equal number of cells, A375-ST-TYR-E2i treated or not with tetracycline for EDEM2 induction, was mixed for further analysis. We performed the experiment in duplicate with label swap. The pelleted cells were incubated with 0.005% digitonin buffer (20 mM Hepes, 110 mM potassium acetate, and 2 mM magnesium acetate) for 5 min on ice. Samples were centrifuged 10 min at 1500 rpm, 4 °C, and the supernatant was removed. Proteins from the remaining pellet were extracted with 2% Triton X-100 buffer (2% Triton X-100 [v/v], 150 mM NaCl, 1.5 mM MgCl_2_, and 1 mM EDTA) and then further processed for trypsin digestion as described later. Proteins were precipitated with a solution of 100% trichloroacetic acid, centrifuged at 14,000 rpm, and the supernatant was removed. The pellet was washed with acetone, before drying. Samples were further processed as described elsewhere (29). Briefly, the extracted proteins were solubilized in a chaotropic reagent buffer (6 M guanidine hydrochloride), reduced, and alkylated before ON trypsin digestion. The resulting peptides were desalted on C18 StageTips as described further.

### C18 StageTips Desalting

For peptide desalting, stage tips were used according to previously described protocols (30, 31). Shortly, tips with various numbers of Teflon-immobilized C18 disks were prepared in house. The disks were first activated with 100 to 200 μl methanol and then equilibrated with 100 to 200 μl of the elution buffer (0.5% acetic acid and 80% ACN) and after with 100 to 200 μl binding buffer (0.5% acetic acid). Samples were acidified with acetic acid or by dilution in binding buffer and passed over the C18 disks. The solid material was washed with 100 μl binding buffer and subject to elution by adding twice 75 μl of elution buffer. The eluted peptides were vacuum concentrated and kept at −20 °C until further use.

### Sucrose Density Fractionation

A375-E2 melanoma cells were lysed in 1% digitonin lysis buffer, on ice, for ~30 min, and the lysates were cleared out by centrifugation at 14,000*g* for 20 min. The protein content was loaded on top of a continuous 10 to 40% sucrose density gradient (14 fractions) with a 60% sucrose cushion, followed by ON centrifugation at 30,000 rpm, 4 °C in an SW41 Ti rotor (Beckman). Collected fractions were subjected to trichloroacetic acid precipitation, respectively incubation of sample with 100% trichloroacetic acid solution 4:1 (v:v) ratio for 20 min, pelleted at 14,000*g*, and the obtained pellet was washed three times with ice-cold acetone. The protein sediment was reconstituted in 6 M guanidine hydrochloride buffer, reduced, alkylated, and subjected to ON digestion with trypsin. The resulting peptides were desalted on StageTips, vacuum concentrated, and stored at −20 °C until further analysis.

### nanoLC–MS/MS Analysis

Samples were reconstituted in solvent A (0.06% FA and 2% ACN) and analyzed using an Easy-nanoLC II system (Thermo Scientific) coupled online to a Linear Trap Quadrupole-Orbitrap Velos Pro instrument (Thermo Scientific). For peptide separation, either a 120 min (affinity enrichment samples) or 240 min gradient of 2 to 30% solvent B (0.06% FA and 80% ACN) was used and applied to a C18 RP Acclaim PepMap 100 analytical column. For data acquisition, a data-dependent method was used, with an initial survey scan acquired at 60,000 resolution (*m*/*z* 400) in the Orbitrap, followed by the MS/MS scans of the top five or ten most intense ions from the initial scan, acquired in the linear trap quadrupole. Only charged states of +2, +3, and higher were considered for collision-induced dissociation fragmentation. For glycopeptide analysis, the instrument was set to acquire the data in “high–high” mode with an initial survey scan at 30,000 resolution (*m*/*z* 400) acquired in the Orbitrap and the fragmentation of the top ten most intense ions in the higher collisional dissociation (HCD) collision cell, followed by Orbitrap detection at a resolution of 7500 at *m*/*z* 400. To further increase the mass accuracy, the lock mass mode was enabled using the ion at 445.120026. For the nano electrospray ionization source, the spray voltage was set to 1.8 to 2.0 kV and 275 to 300 °C for the transfer capillary temperature. Dynamic exclusion was enabled with a repeat count of 1 and duration of 30 or 60 s and an exclusion list size of 500. For experiments with multiple injections, an exclusion list was used for the subsequent injection(s) to increase the number of identifications. In general, the order of sample injection was random.

### Experimental Design and Statistical Rationale Analysis

Overall, considering technical and biological replicates, we analyzed 151 samples. Three biological replicates for the affinity enrichement (AE) data (three controls and three samples with six fractions each), biological triplicates (with 2–4 technical replicates) for the glycoproteomics dataset, biological duplicates with label swap for the pSILAC experiment (including also three technical replicates), and biological duplicates with duplicate injections for the sucrose fractionation proteomics experiment. Statistical analysis was performed using GraphPad Prism, version 6.00 (GraphPad Software Inc) and R software, version 3.5.3 (Free Software Foundation’s GNU General Public License), assuming normal distributions. Unless otherwise noted, all error bars represent standard error of the mean.

### Database Search Parameters and Acceptance Criteria for Identifications

For peptide identification, the MS-acquired data were analyzed with the Andromeda search engine, integrated into the MaxQuant environment (32), version 1.6.0.1 or version 1.5.3.17, by searching against the human version of the UniProt database (91,484 sequences on June 2015), to which contaminants were added. Trypsin was selected as the protease with a maximum of two missed cleavages. For the first search, a precursor mass tolerance of 20 ppm was used, whereas for the second search, no more than 7 ppm mass deviation was allowed. For linear ion trap–acquired data, the fragment ion mass accuracy was set to a maximum of 0.5 Da. Cysteine carbamidomethylation (+57.01 Da) was selected as a fixed modification. Methionine oxidation (+15.99 Da) and acetylation of protein N terminus (+42.01 Da) were selected as variable modifications. For EndoH deglycoproteome data analysis, the fragment match maximum tolerance was set to 0.02 Da, and in addition, the variable modifications, *N*-acetylglucosamine (HexNAc) and dHexHexNAc were considered on asparagine residues. In addition, for samples with single/multiple digestions, GluC/both GluC and trypsin were selected as protease with a maximum of four missed cleavages (33). The false discovery rate (FDR) was estimated using the target-decoy method by performing a second search against the reversed database (34). The target FDR for database identifications was set to 1% for both peptide spectrum match (PSM) and protein groups if not otherwise noted. For protein identification, the razor protein FDR option was activated by considering each peptide only once in the process. At least one unique or razor peptide was required for protein grouping. For label-free quantification (LFQ), the maxLFQ option was activated (35) for the calculation of the LFQ values with default values for the minimum and average numbers of neighbors and a minimum LFQ ratio count of 1. For LFQ calculations at the protein level, only razor and unique unmodified and modified peptides (with the modifications mentioned earlier) were considered, and the minimum allowed ratio count for protein was 2. The option “stabilize large LFQ ratios” was selected to control protein ratio calculations according to the overlap of the features between the samples. To increase the extracted quantitative information, the match between runs options was also activated for replicates in each group under a match time window of 0.7 min and a time window alignment of 20 min. For a valid match in any pair-wise peptide intensity combination, at least one of the features was required to be identified in MS/MS scans. EDEM2 AE and sucrose density fractionation experiments were searched against the same database using SEQUEST integrated in Proteome Discoverer, version 1.4 (Thermo Fisher Scientific) without any scan threshold, charge filtering, or retention time limit. Searched parameters were similar with those used in MaxQuant Andromeda algorithm: human version of the UniProt database, trypsin as the protease with maximum two missed cleavages, cysteine carbamidomethylation (+57.01 Da) as a static modification, and methionine oxidation (+15.99 Da) as a dynamic modification. The maximum allowed mass deviations were set to 10 ppm for precursor ions and 0.5 Da for ion trap fragmentation data. A second search was performed against the reversed sequences of the database for FDR estimation, and the results were filtered at 1% FDR.

### Downstream Bioinformatics Analysis for Validation and Relative Quantification

*For intensity-based relative quantification of the AE experiments*, LFQ values were exported, and results were first filtered for contaminants and identifications from the reversed database. The LFQ values were log transformed, and protein groups with less than three valid values in either sample replicates were removed. Missing values were replaced by values from a normal distribution with a similar standard deviation and a median close to the instrument detection limit (36). A two-sample *t* test with permutation-based FDR correction was applied. Significant enriched proteins were selected at an estimated FDR of 4% with an S_0_ of 1. For SEQUEST data analysis, only PSMs with a ΔCn of at least 0.05 were further considered and peptides with no more than 5 ppm mass deviation at 1% FDR. Protein group assembly was performed in Proteome Discoverer, version 1.4, by allowing no more than 100 protein references per peptide and a peptide relevance factor of 0.4. For spectral counts analysis, the data were exported from SEQUEST and reformatted for significance analysis of interactome (SAINT) express analysis (37, 38). The analysis was performed with the default options by providing already known protein–protein interaction (CANX and SEL1L). SAINTexpress results were further filtered for an average probability score of 0.89 and Bayesian FDR of 2%.

*For sucrose density gradient data analysis*, spectral counts distribution for each protein group were exported, and the normalized spectral count (NSC) score was calculated as previously described (39).

*For EndoH deglycoproteome analysis*, HCD data were exported for further filtering. Based on the observations of the glycopeptides, HCD fragmentation pattern, and previous characterization of the MaxQuant Andromeda score distribution and localization scores (40), quality thresholds were introduced for reliable identifications. Positive identifications of the peptide sequence were considered only peptide identifications with an Andromeda score of at least 40 and a minimum delta score of 8. For the HexNAc confirmation, only MS/MS fragmentations that contain the diagnostic oxonium ions were further retained and even more, for glycosite localization, we further filtered the results for a localization probability greater than 0.75 and a score difference larger than 5 (41). Ambiguous identifications were manually verified. Relative quantification was performed on tryptic glycopeptides with single sequon. Only identifications with at least three quantification values either in the control or in the treated sample were kept for quantification. Similar to the AE data quantification, missing values were replaced with values from a normal distribution. Statistical analysis of the data was performed using the significance analysis of microarray (42) test with a delta of 0.7 and 720 permutations.

*For pSILAC data*, the evidence table and proteinGroups file from MaxQuant output were analyzed using a custom script, since the identifications and quantification values have to be kept separately for K and R peptides (43). Contaminants reverse identifications, peptides containing both R and K residues, nonlabeled peptides (terminal peptides), and low-scoring identifications were filtered out, and only identifications with a posterior error probability value of 0.067 and lower were further kept for analysis. The MaxQuant reported SILAC ratios, and intensity values were log transformed and median centered. The quantification values were aggregated to protein groups by median, and only records with complete values for all three time points considered were further kept. Entries corresponding to reverse, contaminants, marked as only identified by site and with a Q value >0.01 were filtered out from the final proteinGroups table. For single peptides–based protein groups, only those with a unique sequence marked in the peptides table in the corresponding channel were further kept.

### WB Detection

For EDEM2 AE or its partners detection by WB, the samples were processed as previously described (25), and the elution alongside total lysates, was separated by SDS-PAGE, transferred on nitrocellulose membranes, and incubated with the indicated antibodies.

For protein expression detection, cells were harvested, and equal amount of total protein extracted in 1% Triton X-100 buffer, supplemented with protease inhibitors (Roche), was separated by SDS-PAGE. Separated proteins were transferred on nitrocellulose membranes and probed with the indicated antibodies. The antibodies used for WB detection were HA (11867431001) from Roche, CANX (ab22595), ERLEC1 (ab181166), and tubulin (ab18251) from Abcam, actin (612657) from BD Biosciences, DERL1 (D4443) and EDEM2 (E1782) from Sigma, PDIA4 (sc-135901), SEl1L (sc-48081), tyrosinase (T311) (sc-20035), Hsp90 (sc-13119), protocadherin 2 (PCDH2) (sc-376885), integrin alpha-1 (ITGA1) (sc-271034), and melanoma antigen gp75 (TRP-1) (sc-166857) from Santa Cruz Biotechnology.

### Immunofluorescence

A375-ST-TYR cells transfected with siScr or siEDEM2 were seeded onto coverslips 56 h post-transfection and allowed to adhere for 24 h more. Afterward, the cells were washed with PBS, fixed with 1% paraformaldehyde for 30 min at RT, and permeabilized for 3 min with digitonin 0.005% or 0.1% Triton X in blocking buffer (2% horse serum in PBS). The samples were blocked in 2% horse serum for 2 to 3 h at RT and incubated with the corresponding primary antibodies ON in a humidified atmosphere. Next day, the samples were washed and incubated with secondary antibodies coupled with fluorophores for 30 min at RT and mounted on glass slides. Images were acquired with Zeiss LSM 710 (63×, 1.4 numerical aperture, oil) microscope using Zen software (Carl Zeiss Microscopy Deutschland GmbH). Image processing was performed with Fiji-ImageJ software (the National Institutes of Health), and maximum projection of acquired layers is presented in the figure. Colocalization analysis was performed using the ImageJ JACoP plugin (the National Institutes of Health). The images were split into separate channels and used for threshold processing. Three independent experiments were analyzed for each experiment, and the total number of fields analyzed is indicated in the legends of the figures.

### RT-PCR

Total RNA from A375-ST-TYR melanoma cells, transfected with siScr or siEDEM2, was extracted using the RNeasy Plus mini kit (74134; Qiagen), according to the manufacturer's instructions. RNA purity and concentration was determined at 260 and 280 nm using NanoDrop2000 (Thermo Scientific) spectrophotometer. cDNA was synthesized, having a template total RNA, using SuperScript III reverse transcriptase (1808009; Invitrogen). The cDNA fragments corresponding to EDEM2 and actin were amplified with specific primers (E2 forward: CTACCACGCCTACGACAGCTACCTGG; E2 reverse: CACGCTGTCCT GGAGCACTTCAACCAC) using Taq DNA polymerase (M0285; Neb) according to the manufacturer's instructions. The average of three independent experiments of EDEM2 normalized to actin levels is presented in the figure.

## Results

### EDEM2 Is Associated With Members of ERQC and ERAD Machinery

**Fig. 1:**
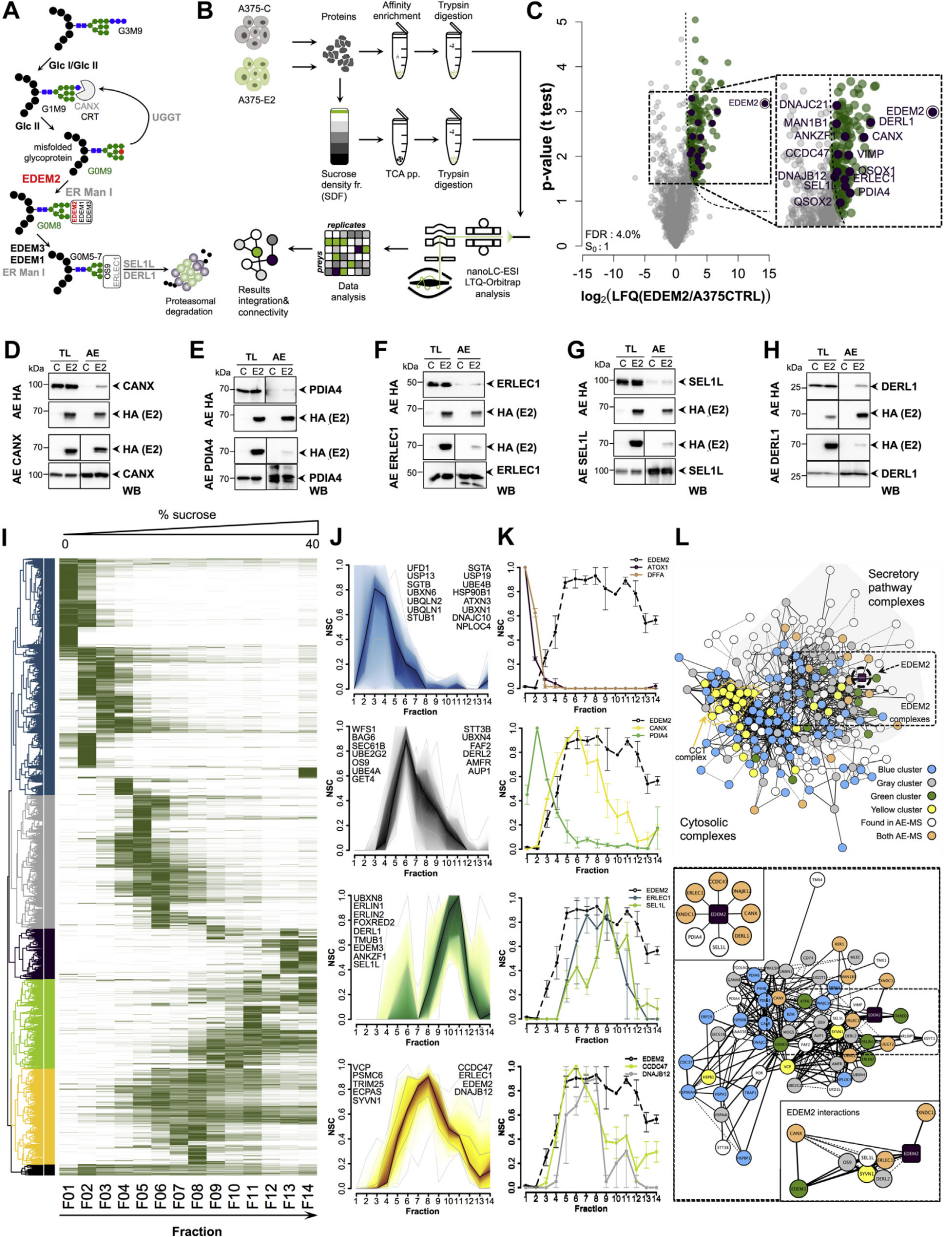
**Analysis of EDEM2-associated proteins in A375 melanoma cells.***A*, schematic diagram of the glycoprotein ERAD (gpERAD). After several unproductive folding attempts in the calnexin/calreticulin cycle (CANX/CRT), misfolded glycoproteins are directed toward gpERAD in which mannose trimming directs the substrate for the final proteasomal degradation. It was proposed that EDEM2 is the first mannosidase in gpERAD by transforming G0M9 to G0M8 glycoprotein structures. *B*, workflow employed for the analysis of EDEM2-associated protein complexes from A375 melanoma cells constitutively expressing EDEM2 HA-tagged (A375-E2). Following protein extraction, samples were AE in EDEM2 or subjected to sucrose density fractionation. AE fractionated proteins were digested, and the extracted peptides analyzed using nanoLC–MS/MS. The results from AE experiments and sucrose density fractionation analysis were annotated from the STRING protein interaction public database. *C*, proteins coenriched with EDEM2 from A375-E2 and A375-C melanoma cells quantified using the MaxQuant LFQ values were designated based on a permutation-based FDR *t* test applied to the differences of log LFQ values between the A375-C and A375-E2 cell lines. Most of the annotated proteins were also statistically enriched at the spectral count level (supplemental Fig. S1*C*). *D*–*H*, WB validation of CANX, PDIA4, ERLEC1, SEL1L, and DERL1 found as proteins coenriched with EDEM2 from A375-E2 melanoma digitonin cell lysates (*upper panels*) and EDEM2 coenrichment from the same lysates using antibodies against the associated proteins (*lower panels*). *I*, hierarchical clustering of proteins identified in sucrose density fractionation based on the NSC profile. Proteins group into multiple clusters according to their fractionation profile (main clusters are shown with different colors). *J*, cluster plot of top four protein clusters (*blue*—*first panel*, *gray*—*second panel*, *green*—*third panel*, and *yellow*—*fourth panel*) for proteins annotated with the ERAD key term in GO UniProtKB database. Shown are the aggregated profiles for the genes mentioned in each panel. It can be observed that EDEM2 coclusters with ERLEC1, CCDC47, and DNAJB12 also found in AE experiments. *K*, fractionation profiles of individual proteins relative to EDEM2. *First panel* denotes unrelated proteins, *second panel* displays PDIA4 and CANX, *third panel* ERLEC1 and SEL1L, and *fourth panel* CCDC47 and DNAJB12, identified as associated with EDEM2. Bars represent SEM of duplicate experiments. *L*, *upper panel*, network analysis of all proteins identified in the AE workflow and those annotated as involved in ERAD or protein folding from the sucrose density fractionation data. Nodes are colored according to the experiment(s) in which were found, and edges width is correlated with the STRING experimental evidence score of the interaction. *Dash lines* denote predicted protein–protein interactions. It can be easily observed that proteins cluster according to the color node identification, and EDEM2 is not associated with proteins from the *blue cluster*. Also the CCT complex found in the *yellow cluster* can be easily identified on the *left side*. In general, proteins clustered according to their subcellular localization (on the *left side* are cytosolic and nuclear proteins and on the right side are secretory-associated proteins). *Lower panel*, close-up view of the EDEM2 containing hub. The *lower inset* shows EDEM2 first neighbors, whereas the *upper inset* denotes proteins found as coenriched with EDEM2 in our experiments. The color coding of the nodes and edges are similar to the ones in the *upper panel*. AE, affinity enrichment; EDEM2, ER degradation–enhancing α-mannosidase-like protein 2; FDR, false discovery rate; GO, Gene Ontology; HA, hemagglutinin; LFQ, label-free quantification; NSC, normalized spectral count; WB, Western blot.

To get insights into the EDEM2-regulated pathway (Fig. 1*A*), we aimed to delineate proteins potentially associated with EDEM2. We used an integrated workflow that combines results obtained from AE and sucrose density fractionation with MS detection (Fig. 1*B*). The AE experiments were performed from digitonin lysates of A375 melanoma cells constitutively expressing empty vector (A375-C) or HA-tagged EDEM2 (A375-E2), and the captured proteins were detected using MS (Fig. 1*B*).

EDEM2 complexes were recovered in 1% digitonin, as we and others have found that most ERAD protein complexes were efficiently extracted in this detergent (25, 44). To designate enriched proteins, experiments were performed in triplicates, and the identified proteins were relatively quantified across conditions using the MaxQuant-reported LFQ values (35). We identified over 36,000 peptides assembled in 3074 protein groups (supplemental Table S1) at 1% FDR (PSM and protein level), quantified with an excellent precision as evidenced from the distribution of the LFQ CV values (supplemental Fig. S1*A*). To assess the significance of enrichment, a *t* test with permutation-based FDR correction was performed using the log of LFQ values. Over 150 proteins (with at least two unique peptides) were found statistically coenriched in the bait sample alongside EDEM2 (Fig. 1*C* and supplemental Table S1). We further annotated the results with semantic terms from Gene Ontology (GO) resource and focused on proteins from ERAD and ER-folding pathways.

As shown in Figure 1*C*, several proteins of these systems were found to be coenriched with EDEM2. For instance, among members of the folding and quality control, EDEM2 was found to be coenriched with CANX, the ER resident protein 70 (PDIA4), DnaJ homolog subfamily B member 12 (DNAJB12), sulfhydryl oxidase 2 (QSOX2) responsible for disulphide bond formation in the secretory pathway (45), TXNDC11, and the folding sensor UGGT2. Some of these proteins, such as CANX or TXNDC11, were previously shown to be associated with EDEM2 (12, 46, 47).

EDEM2 was also found associated with other members of the degradation machinery such as ER mannosyl-oligosaccharide 1,2-alpha-mannosidase (MAN1B1) mainly responsible for glycan trimming of various oligomannosidic structures (48, 49), ERLEC1, SEL1L, and DERL1, all with various roles in ERAD (50, 51, 52). Interestingly, coiled-coil domain–containing protein 47 (CCDC47), a protein responsible for the regulation of ER calcium ions (53), previously shown to be required for the degradation of misfolded α1-antitrypsin and the ER to cytosol dislocation of cholera toxin A1 subunit (54), was also found coenriched together with ankyrin repeat and zinc finger domain-containing protein 1 and VCP-interacting membrane protein, required for the retrotranslocation of misfolded substrates to the cytosol (55, 56). Except for SEL1L, for which we and others have shown its coenrichment with EDEM2 (13, 25), we did not find any reports for their association with EDEM2.

These results were cross checked by an independent analysis of the same dataset performed with a Bayesian-based approach implemented in SAINTexpress (37, 38) (supplemental Fig. S1, *B* and *C* and supplemental Table S2), which relies as input on the abundance of spectral counts of the identified proteins in control and EDEM2 samples. The intersection of the two sets of EDEM2-associated proteins obtained by the aforementioned two statistical approaches resulted in a list of 34 proteins confirmed by both analyses (supplemental Fig. S1*C*), most of them with greater than 15% sequence coverage (supplemental Fig. S1*D*).

The MS results were further corroborated with EDEM2 AE and WB detection experiments. The coisolation of CANX, PDIA4, ERLEC1, SEL1L, and DERL1 (Fig. 1, *D*–*H*, *upper panels*) in EDEM2-enriched digitonin lysates confirmed the initial results. Similarly, EDEM2 was coenriched in reversed-AE experiments (Fig. 1, *D*–*H*, *lower panels*), thus confirming the overall confidence of the dataset.

### Quantitative Proteomics of Density-based Fractionation Protein Complexes Confirms EDEM2-associated Proteins

The AE data suggested a broad heterogeneity of EDEM2 interaction partners, indicating the formation of various molecular entities to exert its function. To characterize the size and composition of these complexes, we have separated them by sucrose density fractionation followed by MS detection and relative quantification. Digitonin lysates of A375 melanoma cells expressing EDEM2 (A375-E2) were subjected to separation on a 10 to 40% continuous sucrose gradient at 30,000 rpm for 17 h in a SW41 Ti rotor. The proteins corresponding to each fraction were precipitated using trichloroacetic acid and subsequently used for MS investigation. Following MS protein identification (supplemental Table S3), we created fractionation profiles for each protein based on its NSC distribution along the gradient (39). Analysis using hierarchical clustering revealed the formation of six main clusters (Fig. 1*I*). As observed in Figure 1*I*, four of these clusters (yellow, green, gray, and blue) enclose more than 90% of the 5287 identified protein groups. The other two clusters encoded proteins with peak apex at very high density (Fr.13 and 14—*dark-purple cluster*) or ones with complexes spread along almost the entire gradient (*black cluster*). Thus, the four main clusters can explain the distribution of most of the protein complexes.

In testing whether the experimental set up is able to capture endogenous protein complexes, we analyzed the fractionation profiles from different clusters and supported this observation using data of previously characterized multiprotein complexes available in the STRING database (57). Supplemental Fig. S2*A* shows the NSC profiles for the beta, gamma, and epsilon subunits of the 14-3-3 protein complex (YWHA-B,-G,-E)—*left panel*, a close-up view of the heat map (*lower panel*) and the STRING annotation of the protein complex (*right panel*). As shown in supplemental Fig. S2*A*, these cocluster at the beginning of the gradient and are found in close proximity on the heat map in the *blue cluster* (*lower panel*), revealing similar NSC profiles. Analysis of the STRING database confirmed this protein complex by finding experimental evidence for protein–protein interactions between all members of the complex (supplemental Fig. S2*A*; *upper-right panel*—edges link all nodes of the complex). Similarly, the NSC profile of several members of the COP9 signalosome complex (COPS7-A, COPS7-B, and COPS8) revealed nearly identical cofractionation and coclusterization in the gray cluster (supplemental Fig. S2*B*). We also noticed cofractionation of members from larger protein complexes, such as the TCP1 chaperonin complex (CCT-2, CCT-4, and CCT-5) (supplemental Fig. S2*C*), estimated to ~850 to 900 kDa (39, 58). Thus, the results captured native protein complexes along the entire gradient.

We next interrogated the sucrose density gradient data for EDEM2-containing complexes and observed their presence in the yellow cluster comprising 823 proteins such as the ERAD members ERLEC1, DNAJB12, and CCDC47 (Fig. 1*J*, *fourth panel*), which were also found to be associated with EDEM2 by the AE experiments as shown previously. Moreover, this cluster also contained two other components of the ERAD ubiquitination machinery (the E3 ubiquitin–protein ligase synoviolin—SYNV1 and E3 ubiquitin/ISG15 ligase TRIM25) and proteins involved in the final steps of ERAD such as valosin-containing protein, the proteasome adapter, and scaffold protein ECM29–ECPAS, which couples the 26S proteasome to the endosomal components (59) and one of the 26S proteasome regulatory subunits—PSMC6. Of note, TRIM25, valosin-containing protein, and PSMC6 were also identified in the AE experiments, although not statistically enriched, suggesting that EDEM2 could have also indirect contacts with members involved in ERAD final steps which supports the notion of proteasome coupling to the ER for misfolded substrate clearance. Proteins from this cluster peak at fractions 6 to 10 with a tailing shoulder in late fractions, similar to the CCT complex, suggesting comparable high-density entities. A similar distribution was found for the endogenous EDEM2, analyzed from digitonin lysates of A375-C, with a maximum overlayed with ERLEC1 and SEL1L, confirming the association with these proteins (supplemental Fig. S2*E*). We further analyzed proteins annotated with the ERAD or folding key terms and found numerous proteins in the adjacent green cluster, which peaks at fractions 8 to 11, comprising slightly higher density protein complexes compared with the ones from the yellow cluster. This included the complex of ER lipid raft–associated proteins 1 and 2 (ERLIN1 and ERLIN2), EDEM3 as another member of the mannosidase-like protein family (60, 61) and also SEL1L and DERL1 similarly found in the AE experiments. Interestingly, the other two main clusters (*blue* and *gray*) did not contain any ERAD members found significantly coenriched in the AE experiments, thus supporting the idea that EDEM2 can establish direct or indirect contacts in various complexes, mainly from the two high-density clusters. Similarly, we found that CANX coclustered with EDEM2 in the yellow cluster from the proteins involved in folding (supplemental Fig. S2*D*).

We also analyzed individual protein fractionation profiles and found that CANX, ERLEC1, SEL1L, CCDC47, and DNAJB12 overlap with EDEM2 (Fig. 1*K*; *second*, *third*, *and fourth panel*s). As a general rule, proteins involved in folding tend to associate in different complexes than those involved in ERAD, usually of lower density (Fig. 1*J* and supplemental Fig. S2*D*). We generally observed that proteins from the blue cluster do not overlap with EDEM2 or only partially overlap (Fig. 1*K*, *first panel*).

The results from EDEM2 coenrichment experiments and sucrose density fractionation were mapped onto the STRING database. Figure 1*L* (*upper panel*) displays as nodes all the proteins found coenriched with EDEM2 and the ones annotated as ERAD or folding components in the GO database from the sucrose fractionation. The edge width maps the STRING experimental evidence score for the interaction between the nodes, whereas the dashed lines denote predicted associations. Color node was mapped according to the cluster identity of the protein from the sucrose density experiments, and the layout was based on the hierarchical clustering of significant GO semantic terms from the cellular component. As observed, EDEM2 does not have direct contacts with cytosolic or nuclear complexes (blue nodes from the blue cluster in Fig. 1*L*, *upper panel*). EDEM2 primary neighbors were found to be either proteins from the yellow cluster (SYNV1 and CANX) or found in AE experiments like SEL1L, ERLEC1, or TXNDC11 (Fig. 1*L*, *lower panel* and *lower inset*). By contrast, no evidence in the STRING database was found for the EDEM2 association with PDIA4, DERL1, CCDC47, or DNAJB12, newly identified associated proteins that support the model of positioning EDEM2 at the crossroad between the ERQC and ERAD systems in forming distinct molecular complexes with members of the two machineries.

### EDEM2 Targets a Glycosylated Melanoma Antigen Mutant to Proteasomal Degradation

**Fig. 2:**
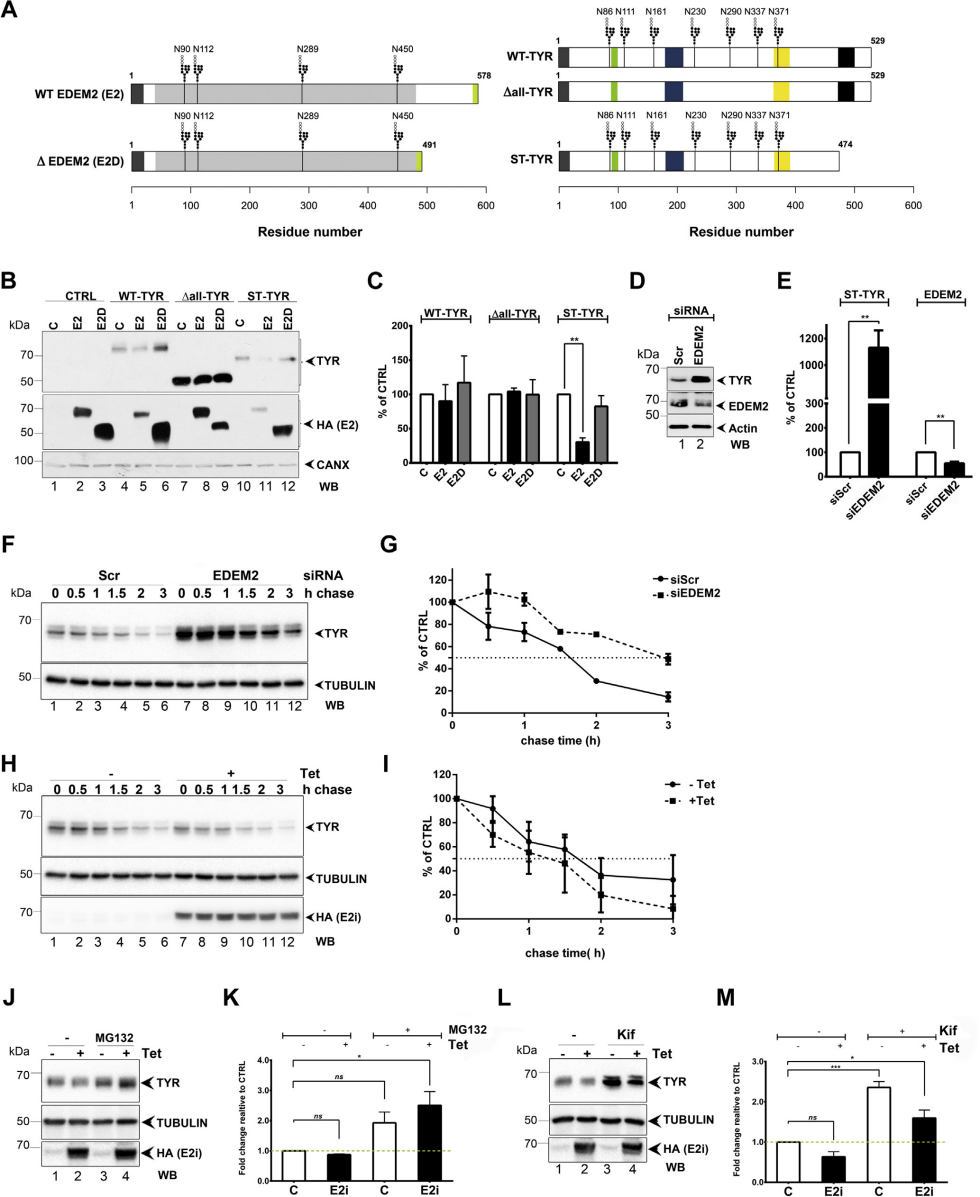
**Functional investigation of EDEM2 in A375 melanoma cells.***A*, sequence representation of the constructs used in A375 melanoma cells: full-length EDEM2 (E2) and EDEM2 missing C terminus (E2D) are depicted in the *left panel*, WT tyrosinase (WT-TYR), nonglycosylated tyrosinase (Δall-TYR), and the construct without the transmembrane and the C-terminal region (ST-TYR) are represented in the *right panel*. The color coding denotes the protein topology: the signal peptide is in *dark gray*, *light gray* denotes the mannosidase domain, and the *light green* symbolizes the HA tag for EDEM2 constructs. For tyrosinase, the signal peptide is in *dark gray*, the transmembrane region in *black*, the epidermal growth factor (EGF)–like domain in *green* and the two copper-binding sites in *dark blue* and *yellow*. For all constructs, *black vertical lines* denote the glycosylation sites. *B*, WB analysis in steady-state conditions of tyrosinase levels in the presence of E2 or E2D. A375 melanoma cells stably expressing each tyrosinase construct (WT-TYR, Δall-TYR, and ST-TYR) or the pLNCX2 vector (CTRL) were transfected with either a mock (C), the full-length EDEM2 (E2), or the C terminus missing EDEM2 (E2D). The protein content from all the samples was subject to SDS-PAGE separation, transferred on nitrocellulose membranes, and incubated with tyrosinase (T311), HA for EDEM2 and E2D detection, and CANX antibodies. *C*, WB bands from *B* were subject to densitometry analysis, and the results were represented as *bar plots*. Bars denote the mean of three biological replicates, and error bars represent SEM. For statistical analysis, a two-way ANOVA with Bonferroni correction was employed (∗∗*p* < 0.01). *D*, WB detection of ST-TYR, endogenous EDEM2, and actin expression level from A375-ST-TYR transfected with siEDEM2 or siScr. *E*, densitometry results of three biological replicates of samples from *D*. Bars represent mean of three biological replicates, and error bars represent SEM. For statistical analysis, a two-tailed *t* test was performed relative to siScr (∗∗*p* < 0.01). *F*, cycloheximide-chase analysis of ST-TYR half-life in A375-ST-TYR cells with downregulated expression of EDEM2. *G*, densitometry analysis of triplicate experiments from *F*. Error bars represent SEM of three replicates. *H*, cycloheximide-chase analysis of ST-TYR half-life from A375-ST-TYR-E2i cell line after EDEM2 induction (E2i) with tetracycline (+Tet). *I*, densitometry analysis of experiments in *H*. Error bars represent SEM of triplicate experiments. *J*, WB analysis of ST-TYR expression levels in A375-ST-TYR-E2i cell line following MG132 treatment. *K*, results in *J* were subject to densitometry analysis: bars represent mean of triplicate experiments, and error bars represent SEM. For statistical significance, a one-way ANOVA, relative to C sample (-Tet/-MG132), with Bonferroni correction was used (∗*p* < 0.05). *L*, similar with *J*, but the cells were treated with kifunensine. *M*, results in *L* were quantified and further subjected to statistical analysis. Bars represent mean of triplicate experiments, and error bars represent SEM. For statistical significance, a one-way ANOVA with Bonferroni correction was used, relative to C (-Tet/-Kif) sample (∗*p* < 0.05 and ∗∗∗*p* < 0.001). EDEM2, ER degradation–enhancing α-mannosidase-like protein 2; HA, hemagglutinin; WB, Western blot.

Previous results have shown that EDEM2 is involved in the degradation of several glycosylated ERAD substrates (7, 10, 11). We previously characterized the soluble version of tyrosinase (ST-TYR) and showed it is an ERAD substrate (21, 22, 23, 62, 63) (Fig. 2*A*, *right panel*). To investigate whether EDEM2 is involved in its degradation, we transiently transfected the full-length EDEM2 (E2) in A375 stably expressing ST-TYR (A375-ST-TYR) and pLNCX2 as control (A375-CTRL) and indeed found a statistically significant lower level of ST-TYR expression compared with the control (Fig. 2*B*, compare lanes 10 and 11, and Fig. 2*C*). By contrast, this was not observed for either WT (WT-TYR) or the nonglycosylated (Δall-TYR) tyrosinase mutant (Fig. 2*B*, compare lanes 7 and 8 and Fig. 2*C*), also a misfolded protein, ER retained, and sent to degradation *via* ERAD (64).

Conversely, an EDEM2 mutant previously found to be nonfunctional (E2D) (10), had no effect upon the stability of WT-TYR, ST-TYR, and Δall-TYR (Fig. 2*B*, compare lanes 6, 9, and 12 with lanes 4, 7, and 10), confirming the role of EDEM2 requirement for its misfolded substrates degradation.

Further validation came from the downregulation of EDEM2 expression using siRNA that resulted in an accumulation of ST-TYR at steady state (Fig. 2, *D* and *E*). The effect was in addition confirmed by an increased half-life of ST-TYR, as shown by cycloheximide chase experiments (Fig. 2, *F* and *G*).

Using a similar experimental design, we analyzed the degradation kinetics of ST-TYR in cells overexpressing EDEM2. For this, a tetracycline EDEM2-inducible expression system in A375 melanoma cells stably expressing ST-TYR (A375-ST-TYR-E2i) was generated. This cell line was characterized regarding EDEM2 expression by titrating the tetracycline concentration and incubation time for various clones, which showed similar time-dependent expression profiles for EDEM2 and ST-TYR (supplemental Fig. S3, *A* and *B*). These recapitulated our initial observations regarding the steady-state reduced expression of ST-TYR following EDEM2 induction (supplemental Fig. S3*B*). As expected, the cycloheximide chase experiment showed that the half-life of the ST-TYR is decreased when EDEM2 expression is activated with tetracycline (Fig. 2, *H* and *I*). These results were corroborated with pulse-chase experiments when cells were labeled with radioactive ^35^S-Met/Cys and chased for specific times (supplemental Fig. S3, *C* and *D*), confirming the EDEM2 dependence of ST-TYR degradation.

Misfolded ERAD substrates are usually sent to degradation *via* a glyco-dependent ubiquitin–proteasome pathway, so we next tested whether proteasome or mannosidase inhibitors would stop the EDEM2-induced degradation of ST-TYR in A375 melanoma cells. When blocking the proteasomal activity, the degradation of ST-TYR was reversed in EDEM2-expressing cells (Fig. 2*J*, compare lanes 2 and 4 and Fig. 2*K*). Kifunensine treatment displayed a similar pattern (Fig. 2*L*, compare lanes 2 and 4 and Fig. 2*M*). Hence, these results suggest that EDEM2 reduces ST-TYR levels in melanoma cells by targeting the substrate to the ubiquitin–proteasome degradation, and this event is dependent on the N-glycan mannose trimming in A375 melanoma cells.

This prompted us to further investigate whether any of the proteins found associated with EDEM2 in the AE could be involved in the degradation of ST-TYR. Therefore, we cotransfected siRNAs targeting SEL1L, ERLEC1, MAN1B1, or DERL1 for 48 h, and afterward cotransfected ST-TYR with mock or EDEM2 and measured the level of each protein by WB detection. The steady-state levels of ST-TYR in mock-transfected samples were slightly upregulated when silencing SEL1L, MAN1B1, and DERL1, but not in siERLEC1 cells (supplemental Fig. S3, *E* and *F*). However, following EDEM2 overexpression, the levels of ST-TYR were considerably rescued compared with the scramble siRNA sample after downregulation of SEL1L, ERLEC1, and DERL1 (supplemental Fig. S3. *E* and *F*), suggesting that EDEM2-induced degradation of ST-TYR is SEL1L, ERLEC1, and DERL1 dependent.

Thus, these results support the hypothesis that EDEM2 is involved in a glyco-dependent degradation of ST-TYR toward the ubiquitin–proteasome system. Alongside EDEM2, several ERAD players found associated with EDEM2, such as SEL1L, ERLEC1, and DERL1, also participate in the ST-TYR degradation process.

### Characterization of the EndoH-sensitive Deglycoproteome and Differential Glycoproteomics of EDEM2 Downregulated Melanoma Cells

**Fig. 3:**
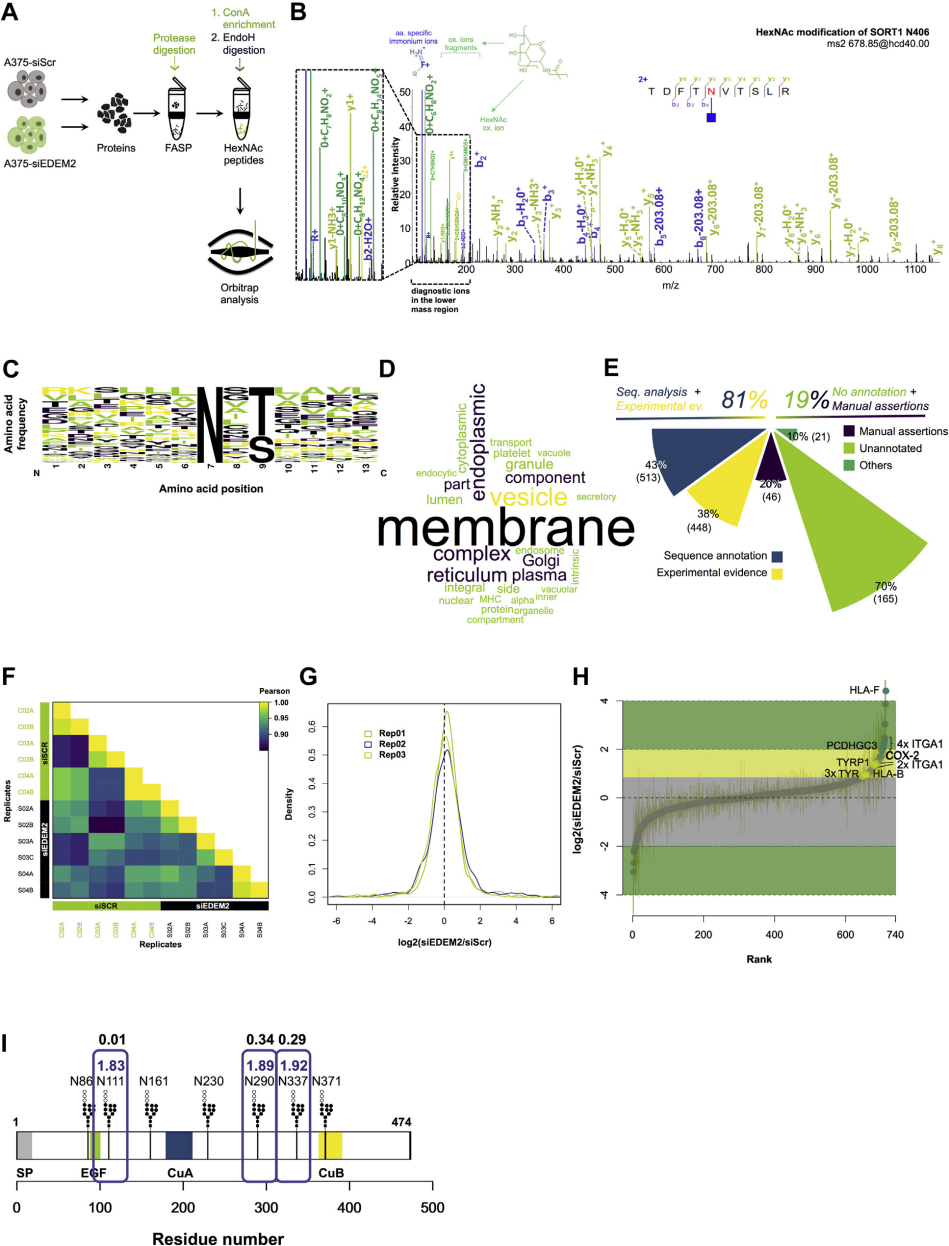
**Analysis of EndoH sensitive deglycoproteome from A375-ST-TYR melanoma cells with downregulated EDEM2 expression.***A*, the workflow of the sample preparation is similar to the classical *N*-glycoFASP, but PNGaseF is replaced with EndoH to release the glycopeptides captured on ConA. *B*, MS/MS fragmentation of the HexNAc modified TDFT[N]VTSLR peptide from SORT1 protein. HCD fragmentation produces both, y ions with the HexNAc residue still attached to the peptide backbone and diagnostic ions visible in the lower mass region of the spectrum for an increased confidence of the identification (*left panel inset*). All ions are detected with high mass accuracy in the orbital trap. *C*, sequence logo annotation with relative frequencies shows that almost all the identified glycosites correspond to the canonical glycosylation motif. A slight preference for threonine relative to serine in the +2 position was observed. *D*, GO analysis of the proteins with identified glycosites shows enrichment for proteins associated with the secretory pathway. *E*, distribution of the identified glycosites relative to the *N*-glycosylation annotation in the UniProtKB database. Almost 81% of the data is annotated in the database; however, only 38% of the glycosites have experimental evidence. The remaining 19% of the glycosites are either nonannotated or annotated as manual assertions of curators. *F*, Pearson correlation coefficient heat map of replicates. It can be observed that the lowest values are close to 0.9, showing good reproducibility between replicates. *G*, density histogram of the ratios of the biological replicates. These are normally distributed and zero centered. *H*, log ratios of siEDEM2/siScr were ranked in an S plot to reveal enriched glycosites between the two samples. Points denote the average of three biological replicates, and error bars represent SEM. Possible candidates were found using SAM analysis. Labels on *green* points designate statistically enriched glycosites, and labels on *yellow* points denote glycosites found with ratios higher than ST-TYR. *I*, schematic representation of ST-TYR denoting the precision of the relative quantification for the analyzed glycosites. Shown are the log ratios for each glycosite quantified, and aforementioned SEM values are denoted. EDEM2, ER degradation–enhancing α-mannosidase-like protein 2; GO, Gene Ontology; HCD, higher collisional dissociation; *N*-glycoFASP, *N*-glyco filter–aided sample preparation; siEDEM2, siRNA sequence targeting EDEM2; siScr, noncoding RNA sequence, scramble.

To identify additional glycoproteins requiring the EDEM2 lectin function for degradation, as found for the ST-TYR protein, we used high-resolution MS and characterized the high mannose and hybrid glycopeptides derived from glycoproteins susceptible to EDEM2-mediated degradation. Using a modified version of the *N*-glycoFASP approach (28), glycopeptides extracted from A375-ST-TYR treated with siRNA targeting EDEM2 (siEDEM2) or a nontargeting RNA sequence (siScr) were isolated using lectin affinity and released by EndoH digestion (Fig. 3*A*). EndoH has the advantage to cleave mostly ER-resident glycoproteins, those *en route* to early Golgi, containing high mannose and hybrid glycan structures (65, 66). It also leaves a HexNAc residue attached to the peptide chain, providing evidence for glycosylation (Fig. 3*B* and supplemental Fig. S4*A*). There are also some exceptions, immature N-glycans left unprocessed occurring in secreted glycoproteins, but they are less abundant, as recently shown for breast cancer cells (67).

We identified over 1100 HexNAc-modified glycosites (supplemental Table S4), mapped on more than 600 proteins, most of these corresponding to the N-linked glycosylation motif N-X-[S/T], where X is not proline (Fig. 3*C*), but some also containing cysteine or valine instead of serine or threonine, further confirming previous observations regarding the N-X-C glycosylation motif (supplemental Fig. S4*B*) (28, 41, 68). This suggests deep coverage of the melanoma EndoH deglycoproteome dataset, further confirmed by the wide dynamic range of the glycopeptides intensities (~five orders of magnitude; supplemental Fig. S4*C*) and by comparing replicate samples (over 70% of the glycosites were completely reproducible; supplemental Fig. S4*D*). Whilst most of the identified glycoproteins (~80%) had either one or two occupied glycosylation sites (supplemental Fig. S4*E*), we also identified some heavily glycosylated proteins, such as protocadherin fat 1, laminin subunit alpha-4, and *N*-acetylglucosamine-6-sulfatase (supplemental Table S4). Further analysis of the dataset revealed that ~81% (961) of the glycosites are already annotated in the UniProtKB database (release April 2019) (69), but only around 38% of these (448 glycosites) are annotated with experimental evidence. Thus, approximately 59% (699 glycosites) were either validated in this study or were not previously annotated in the UniProtKB database (Fig. 3*E*). An interesting aspect is that EndoH can also remove the glycans of some core-fucosylated glycostructures, leaving a FucHexNAc on the asparagine residue. Thus, the data were further searched within the database by considering the FucHexNAc (dHexHexNAc) modification. The analysis confirmed the identification of 42 dHexHexNAc-modified glycosites. Thus, the dataset covered more identified glycosites, compared with other endoglycosidase studies, including some fucosylated structures (supplemental Table S5) (70, 71). Although the glycopeptides were obtained by ConA affinity, we note however, that we cannot totally exclude the possibility that HexNAc or FucHexNAc structures could result following intracellular glycan truncation, as previously suggested (72, 73, 74).

To obtain an overview of the enriched subcellular compartments, we performed a GO analysis using database for annotation, visualization, and integrated discovery (75, 76). Genes expressed at the plasma membrane, ER, Golgi apparatus, or secretory vesicles were found significantly overrepresented (Fig. 3*D*), thus confirming the enrichment of proteins associated with the secretory pathway. The data revealed that most of the identified proteins are non-ER/Golgi residents; hence, these are targets of potential substrates for EDEM2-mediated degradation and were further investigated.

To quantify the relative abundance of EDEM2 clients, two sets of cells, that is, A375 ST-TYR melanoma cells transfected with siEDEM2 and siScr were compared. As shown in Figure 3*F*, the reproducibility and precision of the technical and biological replicates were good with Pearson correlation coefficients of glycosite intensities above 0.9 for most of the cases. This is also evidenced by the zero-centered normal distribution of the glycosite ratios between siEDEM2- and siScr-treated cells for the biological replicates (Fig. 3*G*). We also monitored the differential expression of ST-TYR between siScr- and siEDEM2-treated cells, and the precision and accuracy of the quantification was confirmed, as the median fold change was ~1.89 with a CV of ~2% for the monitored glycosites (Fig. 3*I*). Thus, at least in our hands, these results suggest that the EndoH deglycoproteome reflects the relative changes in the abundance of the ER and early Golgi fraction of glycoproteins and can be investigated using LFQ.

The dataset analysis revealed glycosites enriched in siEDEM2-treated cells from several glycoproteins. For statistical analysis, significance analysis of microarray (42) was applied, and ST-TYR was also used as a reference point to denote enriched glycosites (Fig. 3*H*). We found eight sites from five proteins in this category (Fig. 3*H* and supplemental Table S6). One of the most prominent hits in this aspect is ITGA1, which displayed four increased glycosites in siEDEM2-treated cells. ITGA1 and integrin beta-1 are plasma membrane glycoproteins that function as receptors for collagen and laminins (77). Besides ITGA1, we also found an isoform of the HLA-F, protocadherin gamma-C3 (or PCDH2)—a plasma membrane glycoprotein involved in cell adhesion (78), prostaglandin G/H synthase 2 (COX-2)—an ER protein that functions in arachidonate metabolism and was previously shown to be an ERAD substrate (79, 80) and another member of the tyrosinase protein family, the TRP-1. Thus, besides providing the basis for the experimental validation of over 1000 new glycosites, these results enabled the quantification of the relative abundances of the ER and early Golgi fractions of glycoproteins in siEDEM2-treated cells.

### Analysis of EDEM2 Clients Degradation Kinetics by pSILAC

**Fig. 4:**
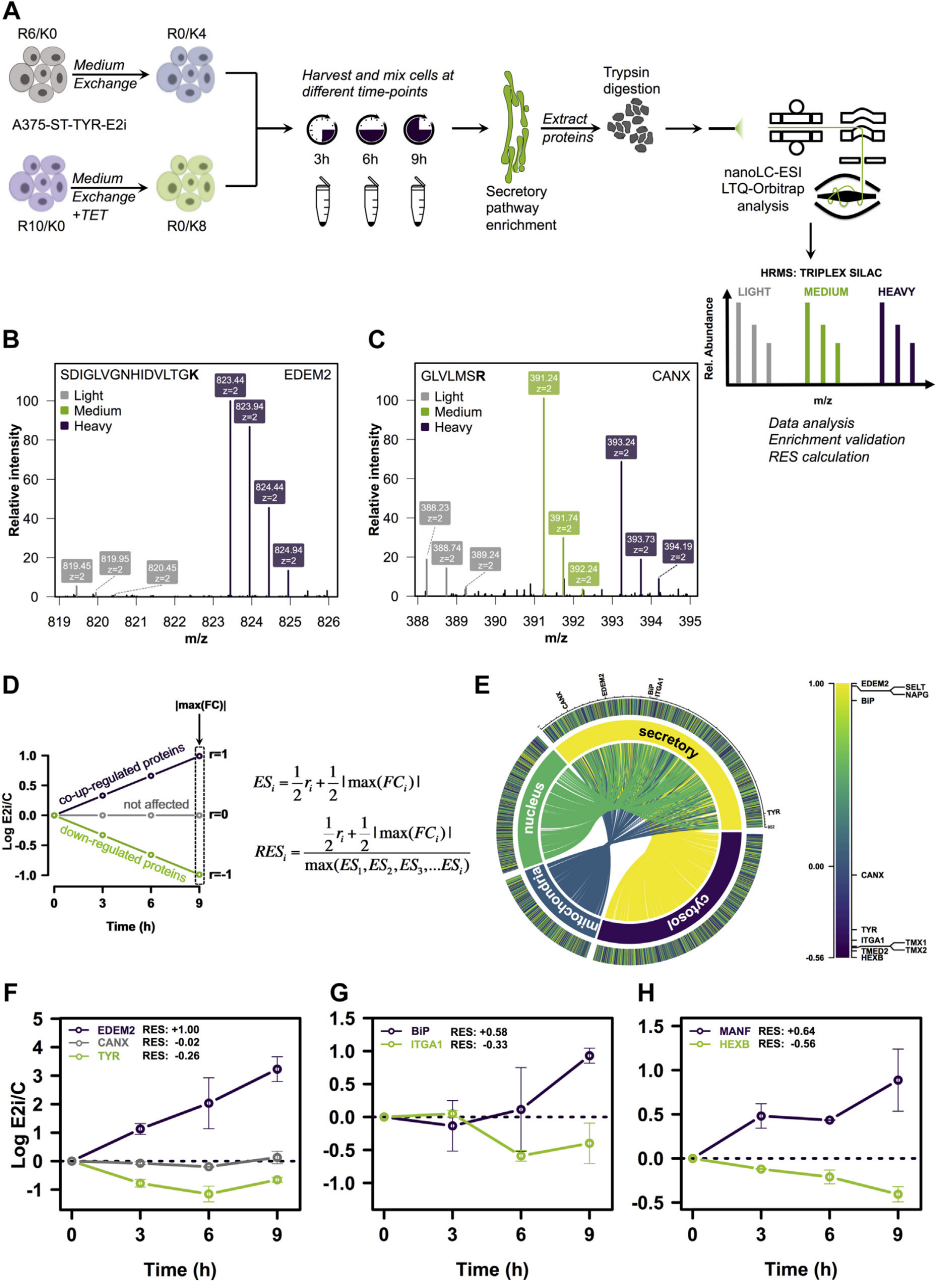
**Validation of EDEM2 candidates in A375-ST-TYR-E2i melanoma cells by pSILAC.***A*, schematic workflow of the pSILAC analysis strategy. A375-ST-TYR-E2i cells were grown in SILAC media containing light K (K0) and labeled R (R6/R10). At the starting of the experiment, the SILAC media were switched to labeled K (K4/K8) and light R (R0). At the same time, tetracycline was added in one of the conditions, which induces EDEM2 expression (E2i). Cells from both conditions were harvested at different time points and mixed. Following digitonin permeabilization, to remove the cytosol content, the extracted proteins were trypsin digested and analyzed by nanoLC–MS/MS. Each peptide will appear as a triplex in the spectrum allowing relative quantification between the samples at each time point. *B*, example of an EDEM2-derived tryptic peptide found only in one of the labeled channels, corresponding to the tetracycline-treated sample. *C*, example of a CANX-derived peptide found in both SILAC channels, M and H. *D*, following EDEM2 induction, proteins can be upregulated, downregulated, or remain unchanged subsequent to their analysis in time. This is described by the Pearson correlation coefficient (*r*). Because *r* does not take into account the fold-change level, RES was calculated, which takes into account *r* and the maximum fold change observed for each protein. *E*, *circular plot* depicting RES calculated for proteins identified on both labeled K channels and their UniProtKB annotation. Because an identified protein can have multiple annotations, these are represented as color-coded links between distinct subcellular compartments. The legend in the *right panel* displays the minimum and maximum RES and some key proteins found either as coupregulated with EDEM2 (BiP and SELT), nonaffected (CANX), or downregulated (TYR and ITGA1). *F*, RES and time evolution of EDEM2, CANX, and ST-TYR. Following tetracycline treatment, the level of EDEM2 increases, but CANX remains largely unaffected. At the same time, the relative level of ST-TYR decreases. This is best reflected by their RES values, as EDEM2 had the maximum RES of 1, CANX had a RES close to 0, and ST-TYR displayed a negative RES. *G*, time evolution and RES of BiP (HSPA5) and ITGA1. Following EDEM2 expression, BiP increases and ITGA1 decreases slightly. *H*, similar to *F* and *G*, but for an entry with a positive RES, upregulated in ER stress and one with a negative score: HEXB. CANX, calnexin; EDEM2, ER degradation–enhancing α-mannosidase-like protein 2; HEXB, beta-hexosaminidase subunit beta; MANF, mesencephalic astrocyte–derived neurotrophic factor; pSILAC, pulse stable isotope labeling with amino acids in cell culture; RES, relative evolution score; SELT, Selenoprotein T.

To further confirm the results from the *N*-glycoFASP workflow, we performed a SILAC-based pulse analysis (pSILAC) of A375 melanoma cells overexpressing EDEM2. We took advantage of the established EDEM2-inducible cell line (A375-ST-TYR-E2i) and the pSILAC strategy in which within the same experiment, it is possible to perform the pulse for two conditions. This admits the direct measurement of the peptide ratios between the two conditions at defined time points, thus allowing the description of the differences in the protein degradation or synthesis rates (43, 81). A375-ST-TYR-E2i melanoma cells SILAC labeled with either R10 or R6 and K0 were switched to SILAC DMEM containing R0 and K8 or K4 at the start of the experiment. At the same time, one of the samples was treated with tetracycline to initiate EDEM2 synthesis, and cells were collected from both conditions at three time points, that is, 3, 6, and 9 h (Fig. 4*A*) and mixed. Since EDEM2 expression is expected to perturb the pool of molecules from ER, cells underwent digitonin permeabilization to enrich for the secretory pathway proteins (Fig. 4*A*), after titrating the digitonin concentration for the enrichment of the microsomal pool of proteins (supplemental Fig. S5*A*). These were extracted and subjected to trypsin digestion, and the resulting peptides were analyzed by nanoLC–MS/MS. The pSILAC protocol renders the identification of the analyzed peptides under three possible states depending on the degradation and synthesis rate of each protein: light (L), medium (M), and heavy (H) (Fig. 4*A*; supplemental Tables S7 and S8). Figure 4, *B* and *C* displays examples of MS spectra for peptides found on any of the three SILAC channels. Following tetracycline treatment, EDEM2 expression is induced in only one of the samples, and analysis of the H/M ratio of proteins at different time points will result in kinetic profiles in which proteins can be either coupregulated with EDEM2 expression, downregulated, or not affected (Fig. 4*D*). This is best explained by the evolution of tetracycline-induced EDEM2 per control ratios (E2i/C) in time. We defined the relative evolution score (RES) and used it to score the kinetic profiles of proteins following EDEM2 induction (see Experimental Procedures and Supplementary Information details). Since RES incorporates the Pearson correlation coefficient and the fold change observed for a protein, upregulated proteins will have a positive score and downregulated ones a negative value (Fig. 4*D*). We first verified the enrichment quality of the identified proteins and annotated the proteins for their subcellular location according to the UniProtKB database. As can be observed in Figure 4*E* (*left panel*), the highest number of identified proteins are annotated as associated with the secretory pathway (*yellow label*). Furthermore, most of the identified proteins from other subcellular compartments (*e.g.*, cytosol, mitochondria) are also coannotated with terms from the secretory pathways, thus confirming the selectivity for the secretory pathway proteome (see the color-coded links between the secretory and the other compartments). Analysis of the RES results revealed values between −0.56 and +1.00. As expected, following tetracycline treatment, EDEM2 obtained the highest RES of 1.00, reflecting the anticipated positive trend (Fig. 4*E*, *right panel*). Figure 4*F* shows the RES values and the kinetics ratio of E2i/C obtained during 9 h for three model proteins: EDEM2 as an upregulated protein following tetracycline treatment of cells with an RES of 1.00, CANX as a relatively constant protein with an RES close to 0, and ST-TYR with a decreasing ratio and a negative RES of −0.26. Similarly, BiP was found as a coupregulated protein with a RES of +0.58 (Fig. 4*E*, *right panel* and Fig. 4*G*) and ITGA1 as a downregulated protein with an RES of −0.33 (Fig. 4*E*, *right panel* and Fig. 4*G*). The lowest calculated RES was found for an entry corresponding to the beta-hexosaminidase subunit beta ~ −0.56 (Fig, 4*E*, *right panel* and Fig. 4*H*), a lysosomal enzyme found as a cisplatin-regulated protein in melanoma cells (82). Interestingly, mesencephalic astrocyte–derived neurotrophic factor was also found as coupregulated, a protein involved in unfolded protein response by association with the nucleotide-binding domain of ADP-bound BiP, stabilizing the client protein–BiP interaction (83, 84). Other coupregulated effectors found were ER proteins, such as thioredoxin reductase–like selenoprotein T or gamma-soluble NSF attachment protein, involved in the vesicular transport between ER and Golgi according to UniProtKB, with scores of ~0.73 (supplemental Table S7). Among the downregulated proteins, we noticed transmembrane emp24 domain–containing protein 2 involved in protein vesicular trafficking (RES ~ −0.4) and thioredoxin transmembrane members 1 and 2 (TMX1 and TMX2), both with RES close to −0.37 (supplemental Table S7). Thus, the results not only confirmed ST-TYR and ITGA1 as EDEM2 candidate substrates in melanoma cells but also revealed a molecular picture of proteins sensitive to EDEM2 expression. However, the pSILAC workflow did not render sufficient proteome coverage to validate all our initial candidates, so these were further assessed by alternative methods.

### Validation of Potential EDEM2 Endogenous Substrates in Melanoma Cells

**Fig. 5:**
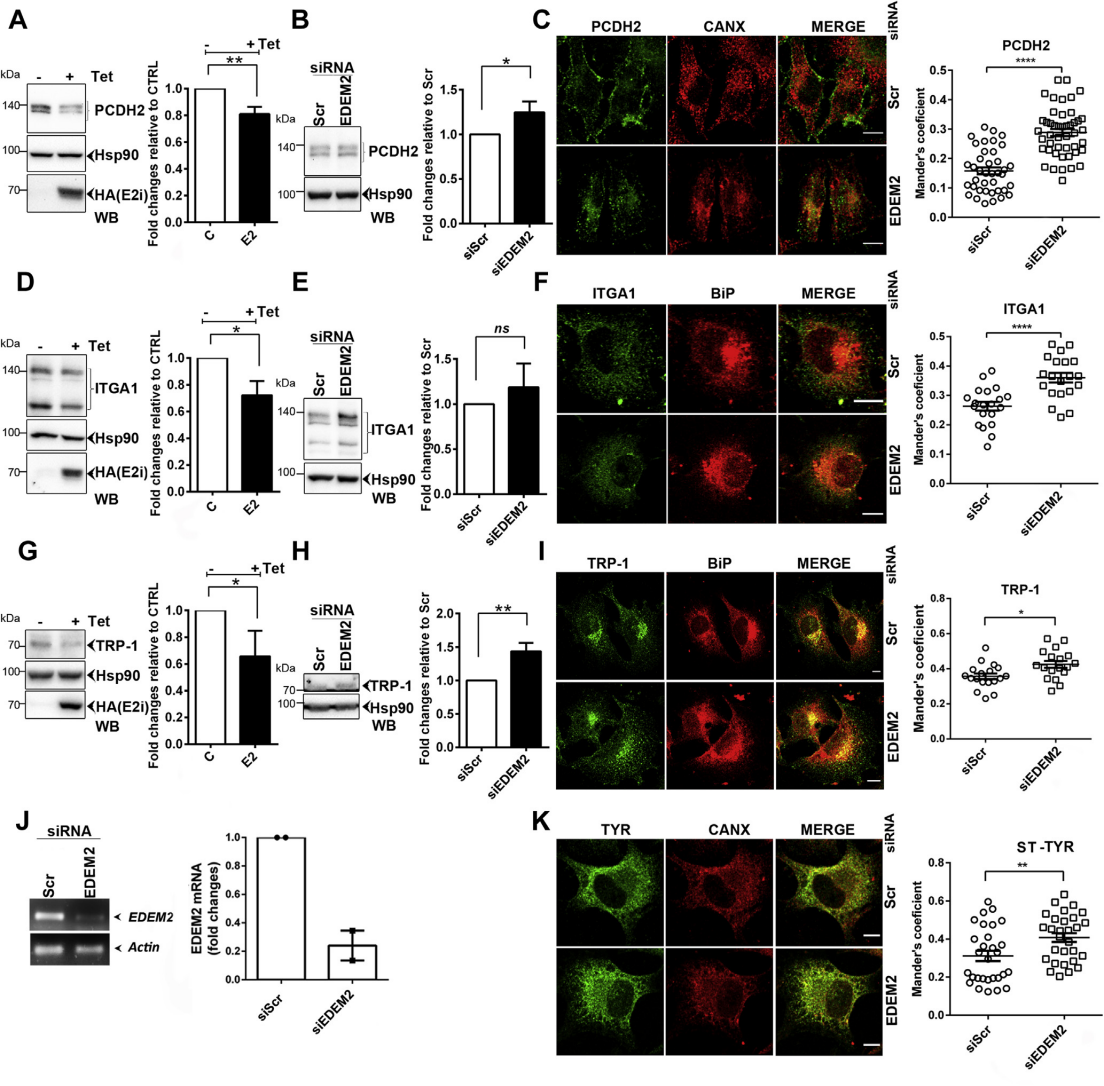
**Analysis of EDEM2 candidate substrates in A375 melanoma cells.***A*, *left panel*, expression level of PCDH2, a glyco-FASP EDEM2 candidate, in A375-ST-TYR-E2i melanoma cells. Cells were treated or not with tetracycline 24 h for EDEM2 induction, harvested, lysed in Triton X-100 buffer, separated by SDS-PAGE, transferred on nitrocellulose membranes, and probed with PCDH2 antibodies, Hsp90 for loading control, and HA for EDEM2 detection. *Right panel*, densitometry analysis of experiments from *A*; bars represent mean of triplicate experiments, and error bars represent SEM. *B*, *left panel*, expression level of PCDH2, in A375-ST-TYR melanoma cells with downregulated expression of EDEM2. The A375 ST-TYR cells were transfected with siScr or siEDEM2, harvested, lysed in Triton X-100 buffer, separated by SDS-PAGE, transferred on nitrocellulose membranes, and probed with PCDH2 antibodies and Hsp90 for loading control. *Right panel*, densitometry analysis of experiments from *B*. *C*, colocalization of PCDH2 with the ER marker CANX in A375-ST-TYR cells transfected with siScr (*upper panels*) or siEDEM2 (*lower panels*) was evaluated. Images for PCDH2 costained with antibodies for CANX are presented; the scale bar represents 10 μm. Colocalization of PCDH2 with CANX, evaluated by calculating Mander's correlation coefficient using the JACoP plugin (mean n = 40 ± SEM). *D* and *E*, *left panels*, expression level of ITGA1 in A375-ST-TYR-E2i melanoma cells (*D*) and A375-ST-TYR treated with siScr or siEDEM2 (*E*) processed as in *A* and *B*. *Right panels*, densitometry analysis of experiments in *D* and respectively *E* for ITGA1. *F*, colocalization of ITGA1 with the ER marker BiP in A375-ST-TYR cells transfected with siScr (*upper panels*) or siEDEM2 (*lower panels*). Confocal images for ITGA1 and BiP are presented; the scale bar represents 10 μm. Colocalization of ITGA1 and BiP was evaluated by calculating Mander's correlation coefficient using the JACoP plugin (mean n = 20 ± SEM) and statistical analysis as in *C*. *G* and *H*, *left panels*, expression of TRP-1 evaluated as in *A* and *B*. *Right panels*, densitometry analysis of bands presented experiments in *G* and *H*. *I*, images for TRP-1 costained with antibodies for BiP are presented; the scale bar represents 10 μm. Colocalization of TRP-1 with BiP was evaluated using the JACoP plugin to calculate the Mander's correlation coefficient (mean n = 18 ± SEM), with the same statistical analysis as in *C*. *J*, the level of EDEM2 transcripts in siEDEM2- and siScr-treated cells was determined by PCR (*upper panel*), revealing a downregulation level greater than 60% (*right panel*). *K*, images for ST-TYR costained with CANX. Colocalization was evaluated using the JACoP plugin to calculate the Mander's correlation coefficient (mean n = 29 ± SEM) and the same statistical analysis as in *C*. For all statistical analysis, an unpaired two-tailed *t* test was used (∗*p* < 0.05, ∗∗*p* < 0.01, ∗∗∗*p* < 0.001, and ∗∗∗∗*p* < 0.001). EDEM2, ER degradation–enhancing α-mannosidase-like protein 2; FASP, filter–aided sample preparation; HA, hemagglutinin; ITGA1, integrin alpha-1; ns, nonsignificant; PCDH2, protocadherin 2; siEDEM2, siRNA sequence targeting EDEM2; siScr, noncoding RNA sequence, scramble; TRP-1, melanoma antigen gp75.

To confirm some of the identified potential substrates, we monitored the candidates, PCDH2, ITGA1, and the melanoma antigen TRP-1, expression, and intracellular distribution in A735-ST-TYR melanoma cells with either overexpressed or downregulated EDEM2 protein expression. As observed in Figure 5, *A*, *D*, and *G* (*left panels*—WBs and *right panels*–quantification), the expression of all three proteins is decreased when EDEM2 is overexpressed. The specificity of the observed effect was also supported by an increased protein level when EDEM2 was downregulated, as shown in Figure 5, *B*, *E*, and *H* (*left panels*—WBs and *right panels*—quantification).

Moreover, ER accumulation of proteins was corroborated with colocalization of candidates with ER markers using confocal microscopy in EDEM2 knocked-down cells. As observed in Figure 5, PCDH2 (Fig. 5*C*), ITGA1 (Fig. 5*F*), and TRP-1 (Fig. 5*I*) are retained in the ER following cells treatment with siRNA targeting EDEM2. These observations are further sustained by an increased colocalization with the ER markers CANX (Fig. 5*C*) and BiP (Fig. 5, *F* and *I*) as shown by significantly increased values for the corresponding Manders's coefficient (Fig. 5, *C*, *F*, and *I*, *right panels*). We verified the silencing of EDEM2 by RT-PCR, and all samples showed roughly 60% decrease in EDEM2 mRNA level (Fig. 5*J*). Similarly, an increased colocalization of ST-TYR, shown earlier to be a substrate for EDEM2, with the ER marker CANX was observed in cells transfected with siEDEM2, also evidenced by the increased Manders's coefficient (Fig. 5*K*, *right panel*). Thus, considering all these results, we show here that, at least in our hands, in A375 melanoma cells, ITGA1, PCDH2, and TRP-1 are phenotypically similar with the novel EDEM2 substrate, ST-TYR.

## Discussion

The aim of this study was to characterize the functional proteomic landscape of EDEM2 and define its endogenous candidate substrates in melanoma cells. We find a network of ER-resident proteins that associates with EDEM2 in distinct protein complexes and describe a number of potential EDEM2 substrates involved in melanoma adhesion and progression.

AE–MS approach revealed potential new players, such as DERL1, ERLEC1, DNAJB12, CCDC47, UGGT2, MAN1B1, or QSOX2 in the EDEM2-mediated selection and degradation of ER-misfolded glycoproteins. These proteins that are all ER residents, could act independently of EDEM2 but occurring in its proximity, or could participate in conjunction with EDEM2 in ERQC and ERAD machineries to regulate glycoprotein folding and degradation. To discriminate between these possibilities, the AE–MS approach was complemented with the analysis of EDEM2 protein complexes separated by sucrose-gradient ultracentrifugation. Similar to previous findings (13, 25, 47), we show here that EDEM2 is indeed associated with CANX and SEL1L. This is consistent with results of the sucrose-density sedimentation experiments indicating that EDEM2 can assemble in the same degradation complex together with other gpERAD members, such as ERLEC1, DNAJB12, or CCDC47, forming complexes resolved as different sedimentation species than the ones formed by other proteins, such as PDIA4, DNAJC10, or HSP90B1. Thus, at least in our hands, EDEM2 is associated with members of both the ERAD pathway and the quality control system in melanoma cells. These observations suggest that EDEM2 is positioned at the crossroad between the ERQC system and the ER degradation machinery. This is in agreement with the hypothesis that EDEM2 is the first mannosidase to act in gpERAD of misfolded glycoproteins, as this position would require interactions with both folding and degradation players. Nonetheless, its interaction with DERL1 does not exclude the positioning of EDEM2 in the early retrotranslocation steps of the ERAD substrates (52, 85).

It is important to note that we cannot rule out the possibility of EDEM2 being recognized within the ER as an ERAD substrate and thus displaying valid interactions from this position, since we cannot discriminate between direct or indirect protein–protein interactions. However, the substantiation of ST-TYR as an EDEM2 degradation substrate in two separate approaches involving either overexpression or downregulation of EDEM2 in melanoma cells validates the results of the AE proteomic approach. Furthermore, the modulation of ST-TYR turnover by EDEM2 together with the inhibition of degradation by proteasome inhibitors reinforce the hypothesis that EDEM2 induces degradation of ST-TYR in the ubiquitin–proteasome pathway and that this degradation could be at least partially mediated by the EDEM2 protein complexes, as the siRNA experiments of ERLEC1, DERL1, and SEL1L suggest.

By applying a customized glycoproteomic workflow, we have mapped new glycosylation sites and also characterized for the first time the EndoH-sensitive deglycoproteome of melanoma cells. The EndoH deglycoproteome of EDEM2 knock-down melanoma cells is of special interest considering that this fraction includes EDEM2 endogenous substrates accumulating within the ER (1, 86, 87). This is well illustrated by not only the various canonical EDEM2 substrates such as the null-Hong-Kong mutant alpha1 antitrypsin or beta secretase 476 (10, 11) but also the ST-TYR previously revealed to be an ERAD substrate (21, 22, 62). Here, we show that EDEM2 is involved in ST-TYR degradation. Moreover, there is an increase in the amount of ST-TYR glycosites in the EndoH deglycoproteome analyzed. Furthermore, this allowed us to use the same system to find novel endogenous candidates for EDEM2 in human melanoma cells. The highest glycopeptides accumulated were derived from COX-2, HLA-F or HLA-B, PCDH2, ITGA1, and TRP-1 that are possible novel EDEM2 candidate substrates. Some of these candidates were previously shown to be ERAD substrates, like COX-2 (79, 80, 88), whose degradation is N-glycan dependent. This is in accordance with our data showing the ER accumulation of COX-2 glycopeptides upon EDEM2 downregulation. Similarly, it has been reported that misfolded dimers of the HLA-B27 allotype are ER retained and subjected to degradation *via* ERAD by EDEM1 in a DERL1–dependent mechanism (89). The other novel candidates, TRP-1 and tyrosinase, are known as melanoma antigens processed within the ER (23). Therefore, our results offer new insights into the ERAD process of these antigens and of the major histocompatibility complex I system suggesting a possible role of EDEM2 in the antigen presentation in the context of the HLA complex.

Furthermore, we used pSILAC to analyze the differential protein turnover in melanoma cells upon EDEM2 overexpression. The use of SILAC and MS analysis allowed the generation of comprehensive datasets reflecting the differential kinetic profiles of endogenous proteins. However, a particular advantage of the pSILAC workflow is that the fraction of proteins with short half-life affected by EDEM2 expression (such as the potential misfolded substrates) can be assessed by the E2i/C ratios using the K peptides, and the protein molecules with longer half-life (such as the mature folded polypeptides) can be evaluated using the ratios from R peptides. Analysis of the distribution for E2i/C ratios for each time point for both replicates on the two SILAC channels revealed a slightly larger distribution of K E2i/C ratios compared with the R E2i/C ratios (supplemental Fig. S5, *C* and *D*). This suggests that EDEM2 expression had a larger effect on the newly synthesized fraction of proteins, after tetracycline treatment, than on those of pre-existing molecules (43). Interestingly, the distribution of the K-E2i/C-derived ratios appeared to slightly narrow with increasing time (supplemental Fig. S5*C*), compared with the fairly constant E2i/C ratio distribution found on the R channel (supplemental Fig. S5*D*), suggesting a possible adaptation of cells to EDEM2 expression. Along with ST-TYR, downregulated in the presence of tetracycline-induced EDEM2, a number of other proteins have been identified, including TMX1, TMX2, and also ITGA1, initially found in the EndoH deglycoproteome approach, suggesting these as potential clients of EDEM2-regulated degradation.

For PCDH2, ITGA1, and TRP-1, we were able to validate the results by a complementary approach involving either downregulation or overexpression of EDEM2, followed by SDS-PAGE analysis, WB, and immunofluorescence with specific antibodies. Thus, modulation of EDEM2 expression induces either a decrease or an accumulation of candidate proteins expression, respectively. Moreover, EDEM2 silencing reveals ER accumulation of the three glycoproteins that colocalize with CANX and BiP in the ER. Interestingly, ITGA1 is retained in the ER and targeted to proteasomal degradation by the TRC8 E3 ligase (90, 91, 92). Here, we show that the degradation of misfolded ITGA1 glycoforms involves an EDEM2-related step occurring probably earlier than the ubiquitination step, which facilitates ERAD. PCDH2 and ITGA1 are adhesion proteins with crucial roles in melanoma invasion (93, 94). Thus, it is likely that EDEM2 has a regulatory role in melanoma metastasis and invasion through the regulation of degradation and trafficking of at least these glycoproteins. Thus, the results not only confirm that the misfolded fraction of these proteins is ER retained and sent to ERAD, but even more, their degradation could be dependent on the characterized degradation complexes. Importantly, beside glycosite quantification, the deglycoproteomic workflow also revealed the identification of new glycosylation sites, since ~60% of the identified glycosites are not annotated with experimental information in the UniProtKB database (Fig. 3). This will add valuable information to the N-glycosite Atlas (95) and SAGS glycobase (96, 97), including over 1000 glycosites from this study.

In conclusion, using an integrative proteomic approach, we mapped EDEM2-associated proteins and revealed the main components of the EDEM2-degradation complexes that function in ERAD. We also found canonical and new endogenous candidate substrates for EDEM2 by combining deglycoproteomics and pSILAC analysis. Not least, our experiments validate PCDH2, ITGA1, and TRP-1, as glycoproteins whose expression and intracellular distribution is EDEM2 dependent. These findings will help elucidate the molecular mechanisms of EDEM2 in protein degradation and map several targets in melanoma cells that could finally benefit the translational research field.

## Data Availability

All MS raw data and annotated MS/MS spectra of single peptide identifications have been deposited to the ProteomeXchange Consortium *via* the PRIDE (98, 99, 100) partner repository with the dataset identifier PXD025881. Annotated MS/MS spectra of glycopeptide identifications were deposited to MS-Viewer under the search key edu2xbentm.

## Supplemental data

This article contains supplemental data (21, 43, 101).

## Conflict of interest

The authors declare that they have no conflicts of interest with the contents of this article.
