# Supplementary material for: Affinity Proteomics and Deglycoproteomics Uncover Novel EDEM2 Endogenous Substrates and an Integrative ERAD Network
Source: Mol Cell Proteomics. 2021 Jul 29;20:100125. doi: 10.1016/j.mcpro.2021.100125 (PMC8455867; doi:10.1016/j.mcpro.2021.100125)
Supplement: Supplemental Data and Figures S1–S5 [file mmc2.pdf]

Supplemental material to:

Affinity proteomics and deglycoproteomics uncover novel EDEM2 endogenous substrates and an integrative ERAD network

Cristian V.A. Munteanu[1*], Gabriela N. Chirițoiu[2*], Marioara Chirițoiu[2], Simona Ghenea[2], Andrei-Jose Petrescu[1], Ștefana M. Petrescu[2#]

[1]*Department of Bioinformatics and Structural Biochemistry,*

[2]*Department of Molecular Cell Biology,*

Institute of Biochemistry, Splaiul Independenței 296, 060031, Bucharest, Romania

[*]These authors contributed equally to this work.

[#]Corresponding author:

Stefana M. Petrescu: stefana.petrescu@biochim.ro

(https://orcid.org/0000-0002-4047-0811),

Splaiul Independenței 296, 060031, Bucharest, Romania

Running title: Affinity proteomics and deglycoproteomics of EDEM2 substrates

Figure S1

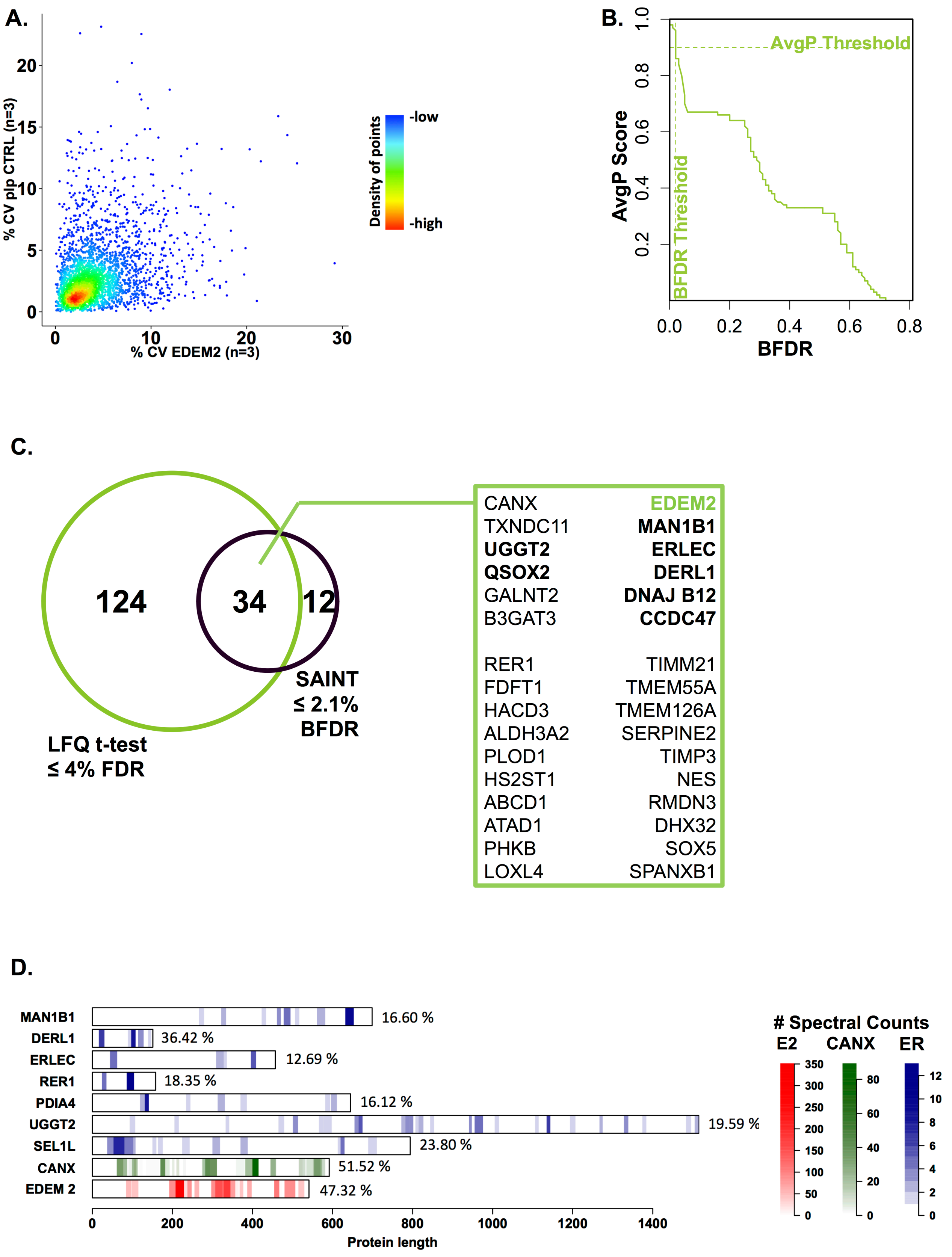

A.
% CV plp CTRL (n=3)
% CV EDEM2 (n=3)
Density of points
-low
-high
B.
AvgP Score
BFDR
AvgP Threshold
BFDR Threshold
C.
124
34
12
LFQ t-test
≤ 4% FDR
SAINT
≤ 2.1%
BFDR
CANX
EDEM2
TXNDC11
MAN1B1
UGGT2
ERLEC
QSOX2
DERL1
GALNT2
DNAJ B12
B3GAT3
CCDC47
RER1
TIMM21
FDFT1
TMEM55A
HACD3
TMEM126A
ALDH3A2
SERPINE2
PLOD1
TIMP3
HS2ST1
NES
ABCD1
RMDN3
ATAD1
DHX32
PHKB
SOX5
LOXL4
SPANXB1
D.
MAN1B1 16.60 %
DERL1 36.42 %
ERLEC 12.69 %
RER1 18.35 %
PDIA4 16.12 %
UGGT2 19.59 %
SEL1L 23.80 %
CANX 51.52 %
EDEM 2 47.32 %
Protein length
Spectral Counts
E2
CANX
ER

Figure S1.

A. Distribution of the CV values for the EDEM2 enriched dataset. The CVs of the LFQ values were calculated for each group in biological replicates. The color code estimates the density of points included in a region of a specific color. **B.** The AvgP and BFDR distribution across EDEM2 enriched dataset. The threshold scores considered for the proteins found as associated with EDEM2 in A375 melanoma cells: AvgP: 0.89 with an estimated BFDR of 2 %. **C.** Comparison of the two data analysis workflows used for the evaluation of EDEM2 protein-protein interactions (using the intensity derived LFQ values and SaintExpress using spectral counts distribution). Bold text denotes new EDEM2-associated protein complexes from ERAD and ERQC. **D.** Sequence coverage and spectral counts distribution of EDEM2 and several proteins found as associated with it. Shown are the gene name of each protein, protein length (# of amino acids) and the position of the identified peptide on the protein's sequence with color coding according to the number of spectral counts associated with the specific peptide.

Figure S2

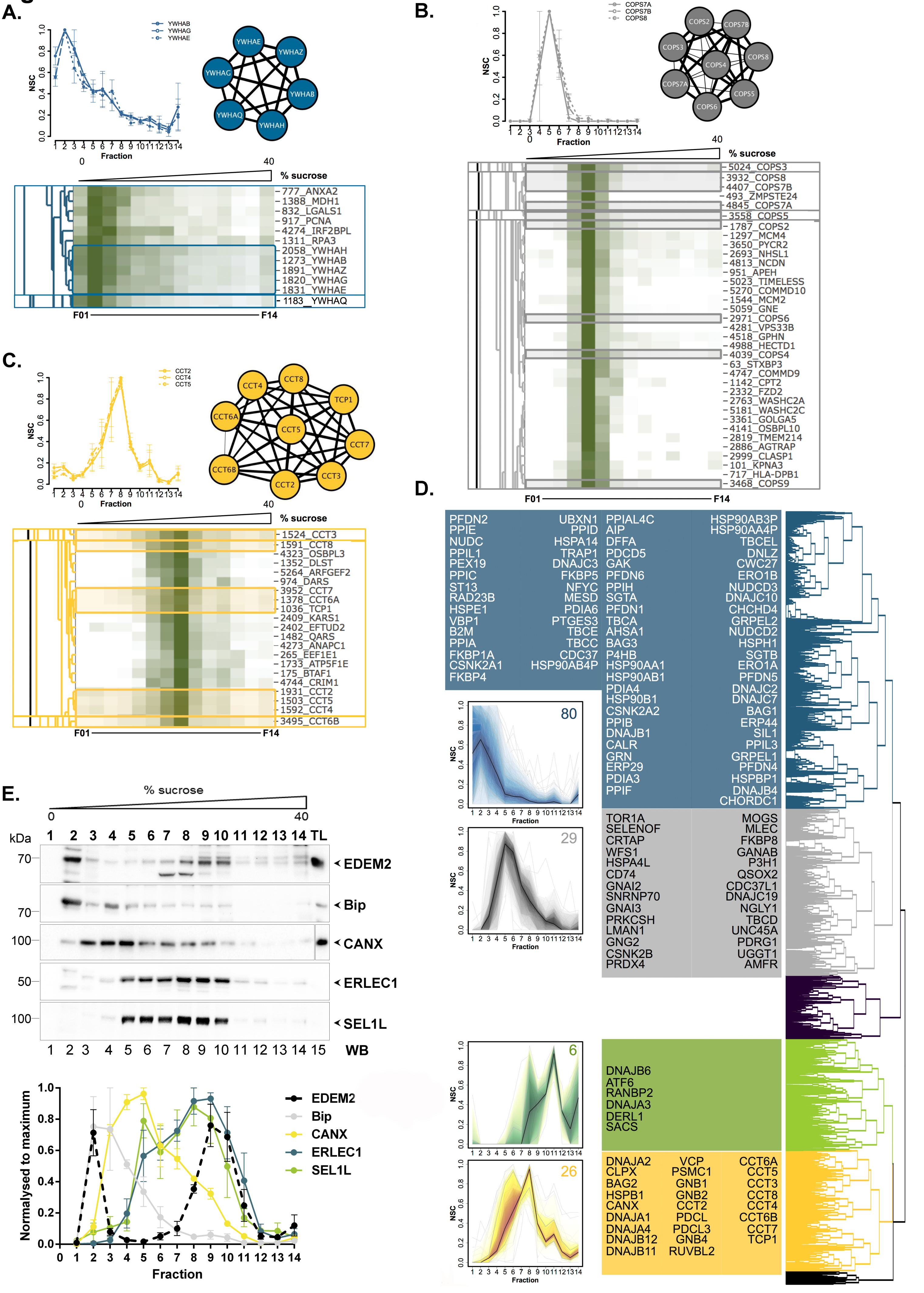
A.
YWHAB
YWHAG
YWHAE
NSC
Fraction
YWHAE
YWHAZ
YWHAG
YWHAB
YWHAQ
YWHAH
% sucrose
0
40
777_ANXA2
1388_MDH1
832_LGALS1
917_PCNA
4274_IRF2BPL
1311_RPA3
2058_YWHAH
1273_YWHAB
1891_YWHAZ
1820_YWHAG
1831_YWHAE
1183_YWHAQ
F01
F14
B.
COPS7A
COPS7B
COPS8
COPS2
COPS7B
COPS3
COPS8
COPS4
COPS7A
COPS5
COPS6
5024_COPS3
3932_COPS8
4407_COPS7B
493_ZMPSTE24
4845_COPS7A
3558_COPS5
1787_COPS2
1297_MCM4
3650_PYCR2
2693_NHSL1
4813_NCDN
951_APEH
5023_TIMELESS
5270_COMMD10
1544_MCM2
5059_GNE
2971_COPS6
4281_VPS33B
4518_GPHN
4988_HECTD1
4039_COPS4
63_STXBP3
4747_COMMD9
1142_CPT2
2332_FZD2
2763_WASHC2A
5181_WASHC2C
3361_GOLGA5
4141_OSBPL10
2819_TMEM214
2886_AGTRAP
2999_CLASP1
101_KPNA3
717_HLA-DPB1
3468_COPS9
C.
CCT2
CCT4
CCT5
CCT4
CCT8
TCP1
CCT6A
CCT5
CCT7
CCT6B
CCT2
CCT3
1524_CCT3
1591_CCT8
4323_OSBPL3
1352_DLST
5264_ARFGEF2
974_DARS
3952_CCT7
1378_CCT6A
1036_TCP1
2409_KARS1
2402_EFTUD2
1482_QARS
4273_ANAPC1
265_EEF1E1
1733_ATP5F1E
175_BTAF1
4744_CRIM1
1931_CCT2
1503_CCT5
1592_CCT4
3495_CCT6B
D.
PFDN2 UBXN1 PPIAL4C HSP90AB3P
PPIE PPID AIP HSP90AA4P
NUDC HSPA14 DFFA TBCEL
PPIL1 TRAP1 PDCD5 DNLZ
PEX19 DNAJC3 GAK CWC27
PPIC FKBP5 PFDN6 ERO1B
ST13 NFYC PPIH NUDCD3
RAD23B MESD SGTA DNAJC10
HSPE1 PDIA6 PFDN1 CHCHD4
VBP1 PTGES3 TBCA GRPEL2
B2M TBCE AHSA1 NUDCD2
PPIA TBCC BAG3 HSPH1
FKBP1A CDC37 P4HB SGTB
CSNK2A1 HSP90AB4P HSP90AA1 ERO1A
FKBP4 HSP90AB1 PFDN5
PDIA4 DNAJC2
HSP90B1 DNAJC7
CSNK2A2 BAG1
PPIB ERP44
DNAJB1 SIL1
CALR PPIL3
GRN GRPEL1
ERP29 PFDN4
PDIA3 HSPBP1
PPIF DNAJB4
CHORDC1
80
TOR1A MOGS
SELENOF MLEC
CRTAP FKBP8
WFS1 GANAB
HSPA4L P3H1
CD74 QSOX2
GNAI2 CDC37L1
SNRNP70 DNAJC19
GNAI3 NGLY1
PRKCSH TBCD
LMAN1 UNC45A
GNG2 PDRG1
CSNK2B UGGT1
PRDX4 AMFR
29
6
DNAJB6
ATF6
RANBP2
DNAJA3
DERL1
SACS
26
DNAJA2 VCP CCT6A
CLPX PSMC1 CCT5
BAG2 GNB1 CCT3
HSPB1 GNB2 CCT8
CANX CCT2 CCT4
DNAJA1 PDCL CCT6B
DNAJA4 PDCL3 CCT7
DNAJB12 GNB4 TCP1
DNAJB11 RUVBL2
E.
% sucrose
kDa
1 2 3 4 5 6 7 8 9 10 11 12 13 14 TL
70
70
100
50
100
EDEM2
Bip
CANX
ERLEC1
SEL1L
1 2 3 4 5 6 7 8 9 10 11 12 13 14 15
WB
Normalysed to maximum
EDEM2
Bip
CANX
ERLEC1
SEL1L
Fraction

Figure S2.
A. First panel: Example of sucrose fractionation profiles for a protein complex found in the blue cluster: 14-3-3 (YWHA) proteins β, γ and ε components. Second panel: STRING annotation of the interaction network for the 14-3-3 complex. Nodes are coloured according to the cluster in which these were found and the width of edges is proportionally with the STRING annotation score from experimentally determined interactions. Third panel: Close-up view of the heatmap region encoding the 14-3-3 protein complex in the blue cluster. **B.** Similar with A. but for a protein complex found in the grey cluster: COP9 signalosome complex with the fractionation profiles for 7A, 7B and 8 subunits (first panel), STRING annotation of the interaction network for the COPS9 signalosome (second panel) and close-up view of the heatmap region encoding the COPS9 signalosome complex in the gray cluster (third panel). **C.** Fractionation profile for the CCT2, CCT4 and CCT5 proteins from the molecular chaperone TRiC complex (first panel), its STRING annotation (second panel) and the heatmap close-up view encoding its components clustered in the yellow cluster (third panel). **D.** Clusterplots of proteins found in the top main four clusters and annotated with the key-term folding in the UniProtKB GO database. Each box denotes the genes found in each cluster and for each the numbers of proteins are represented. It can be easily observed that most of the folding proteins are found in the blue cluster and usually ER chaperons or folding proteins are found in the yellow or green clusters. **E.** Upper-panel: Sucrose fractionation gradient of A375-C cells, with EDEM2 endogenous expression, analysed by WB. Digitonin lysate of A375-C was separated on a 0-40% sucrose gradient and the resulted fractions were precipitated with trichloroacetic acid. Dried pellets were reconstituted in 4% SDS buffer and an equal amount of protein corresponding to each fraction was separated by SDS-PAGE, transferred on nitrocellulose membrane and probed with specific antibodies: EDEM2, for endogenous protein detection, (first panel), BiP (second panel), CANX (third panel), ERLEC1 (fourth panel) and SEL1L (fifth panel). Lower-panel: Graph depicting distribution of endogenous EDEM2, folding and ERAD components in density gradient fractions, after band densitometry of three biological replicates of similar experiments as described in the upper panel. Points are mean of triplicates and error bars are SEM.

Figure S3

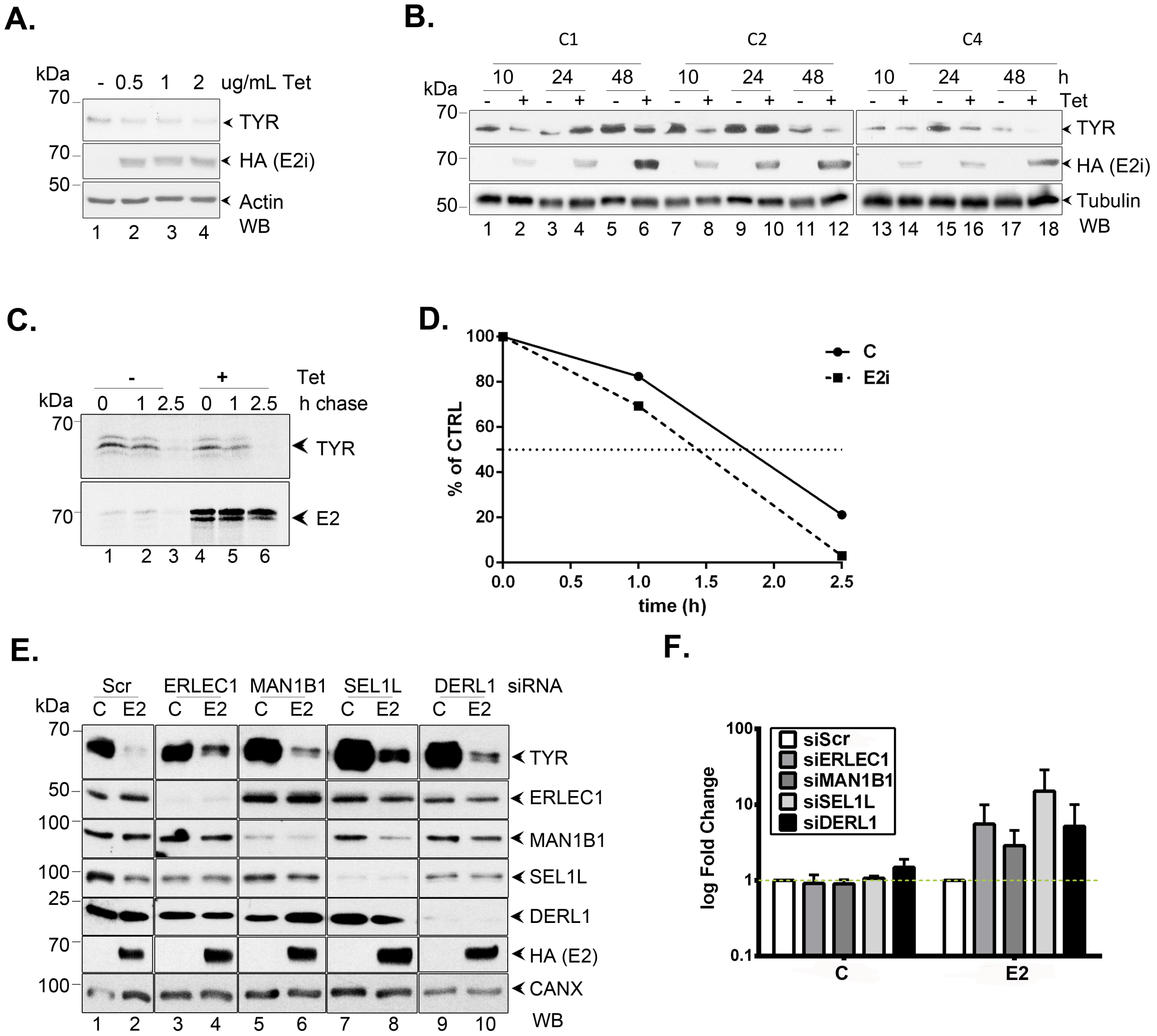
A.
kDa
- 0.5 1 2 ug/mL Tet
70
TYR
70
HA (E2i)
50
Actin
WB
1 2 3 4
B.
C1
C2
C4
10 24 48
10 24 48
10 24 48
h
kDa
- + - + - + - + - + - + - + - + - +
Tet
70
TYR
70
HA (E2i)
50
Tubulin
1 2 3 4 5 6 7 8 9 10 11 12 13 14 15 16 17 18
WB
C.
- +
Tet
kDa
0 1 2.5 0 1 2.5
h chase
70
TYR
70
E2
1 2 3 4 5 6
D.
% of CTRL
100
80
60
40
20
0
0.0 0.5 1.0 1.5 2.0 2.5
time (h)
C
E2i
E.
Scr
ERLEC1
MAN1B1
SEL1L
DERL1
siRNA
kDa
C E2 C E2 C E2 C E2 C E2
70
TYR
50
ERLEC1
100
MAN1B1
100
SEL1L
25
DERL1
70
HA (E2)
100
CANX
1 2 3 4 5 6 7 8 9 10
WB
F.
log Fold Change
100
10
1
0.1
siScr
siERLEC1
siMAN1B1
siSEL1L
siDERL1
C
E2

Figure S3.

A. WB analysis of ST-TYR (TYR), EDEM2 (HA(E2i)), and actin (used as loading control) expression following tetracycline titration (0.5, 1 and 2 μg/mL Tet) in A375-ST-TYR-E2i cell line. **B.** Similar to A., but for three distinct clones of A375-ST-TYR-E2i cell line: (C1, C2, and C4) and different induction times (10, 24 and 48h). **C.** EDEM2 contribution to ST-TYR degradation. A375-ST-TYR-E2i cell line, treated 24 h with tetracycline (Tet) for EDEM2 expression induction, were labelled for 20 min with 35S-Met/Cys and chased for the indicated time points. Cells were lysed in 1% Triton X-100 and immunoprecipitated with anti-tyrosinase and anti-EDEM2 antibodies. The protein A-Sepharose isolated complexes were separated in an 8% polyacrylamide gel, dried and exposed to autoradiography. **D.** Densitometry analysis of bands from C. **E.** Impact of EDEM2 associated proteins (ERLEC1, MAN1B1, SEL1L and DERL1) on the degradation of ST-TYR. 48 h post transfection with the indicated siRNAs (ERLEC (sc-94979), MAN1B1 (sc-92479), SEL1L (sc-61514), and DERLIN1 (sc-60519), all from Santa Cruz Biotechnology), the cells were co-transfected with ST-TYR and mock (C) or EDEM2 (E2). Equal amounts of proteins were separated by SDS-PAGE, transferred on nitrocellulose membranes and probed with the indicated antibodies. **F.** The densitometry results are mean of duplicate experiments represented as barplots with error bars as SEM. Results were normalized to the level of transfected EDEM2 and CANX as loading control.

Figure S4

A.

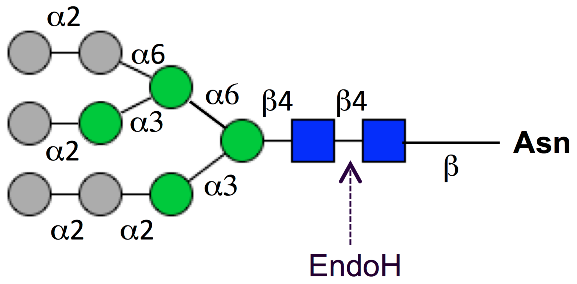

B.

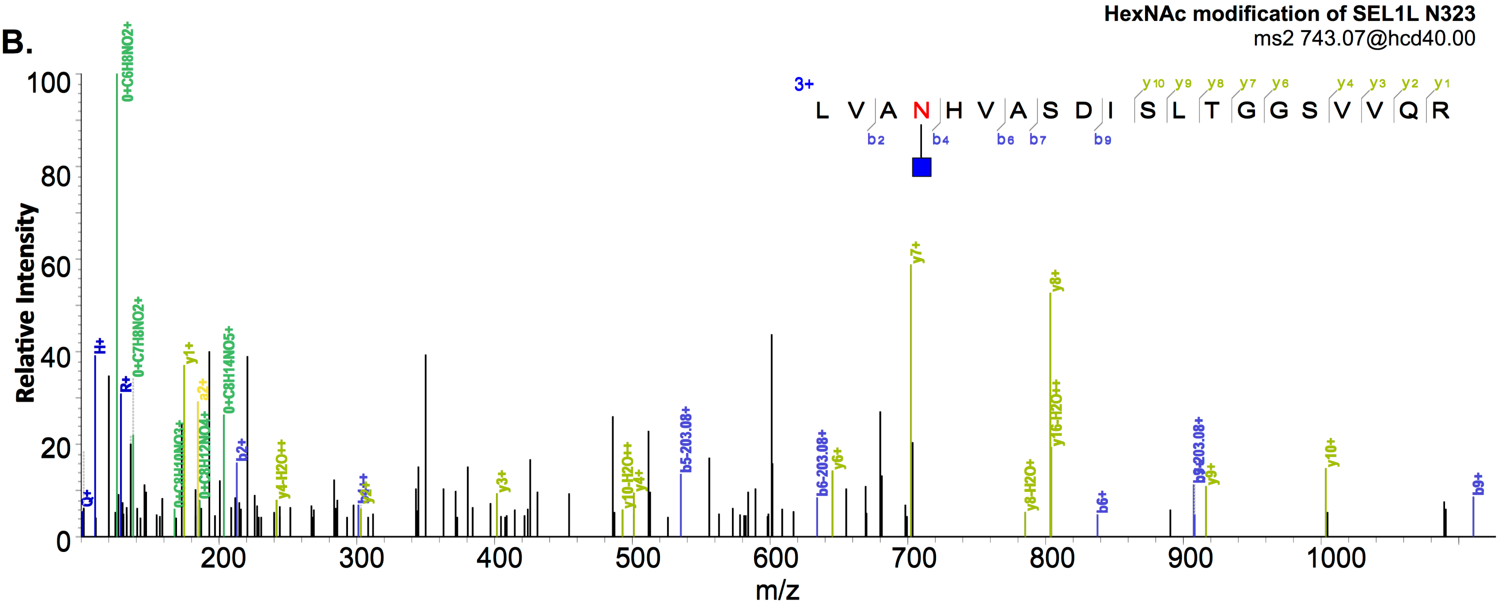

C.

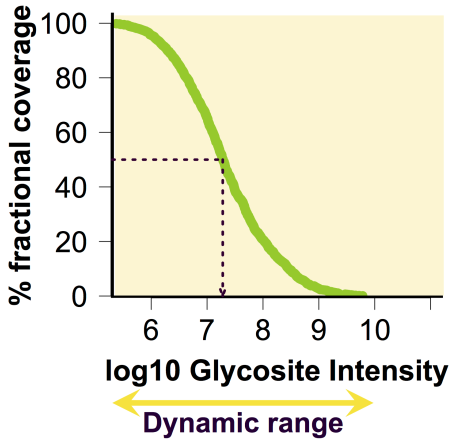

D.

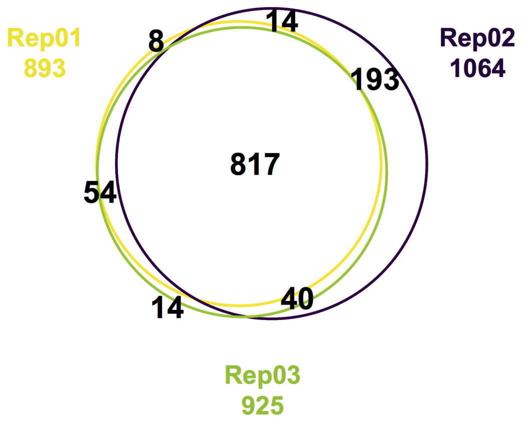

E.

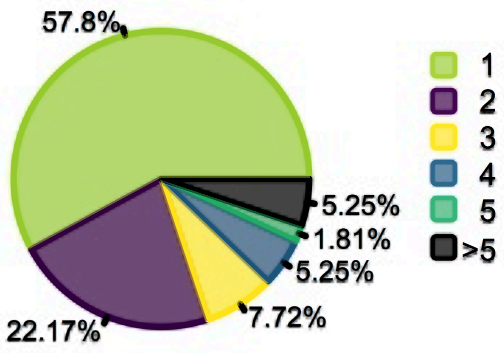

Figure S4.
A. Structure of the high-mannose glycans recognized by EndoH. **B.** HCD MS/MS fragmentation pattern of the SEL1L peptide containing an NXV glycosylation site. **C.** The dynamic range of the EndoH glycoproteome spans almost five orders of magnitude. **D.** VennDiagram of glycosite reproducibility between biological replicates in the control group. **E.** Distribution of the proteins with single or various multiple glycosylation sites identified.

Figure S5

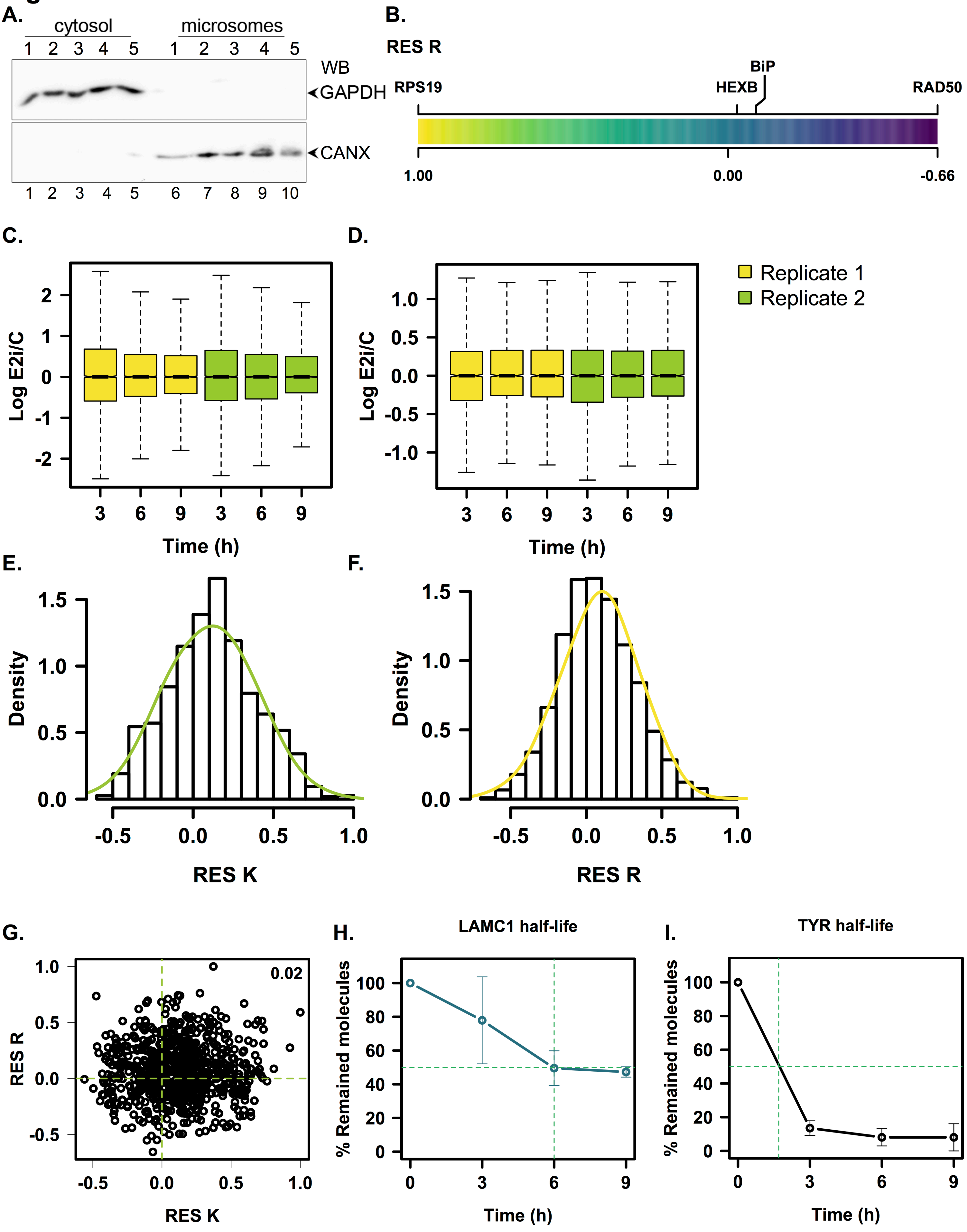

A.
cytosol
microsomes
1 2 3 4 5 1 2 3 4 5
WB
GAPDH
CANX
1 2 3 4 5 6 7 8 9 10
B.
RES R
BiP
RPS19
HEXB
RAD50
1.00
0.00
-0.66
C.
Log E2i/C
2
1
0
-1
-2
3 6 9 3 6 9
Time (h)
D.
Log E2i/C
1.0
0.5
0.0
-0.5
-1.0
3 6 9 3 6 9
Time (h)
Replicate 1
Replicate 2
E.
Density
1.5
1.0
0.5
0.0
-0.5 0.0 0.5 1.0
RES K
F.
Density
1.5
1.0
0.5
0.0
-0.5 0.0 0.5 1.0
RES R
G.
RES R
1.0
0.5
0.0
-0.5
0.02
-0.5 0.0 0.5 1.0
RES K
H.
LAMC1 half-life
% Remained molecules
100
80
60
40
20
0
0 3 6 9
Time (h)
I.
TYR half-life
% Remained molecules
100
80
60
40
20
0
0 3 6 9
Time (h)

Figure S5.
A. Titration of digitonin for cytosolic and microsomal fractionation of A375-ST-TYR-E2i melanoma cells. Five distinct conditions were tested, corresponding to their cytosolic content (lanes 1-5) and microsomal (lanes 6-10). Shown are GAPDH a cytosolic protein and the ER resident protein, CANX. Condition 4 (0.005% digitonin) was selected for further experiments. **B.** RES dynamic range for proteins identified from R-derived peptides. BiP (HSPA5) and HEXB remain relatively unchanged in the nine hours course of time. **C.** Time-dependent distribution of E2i/C ratios for K-labelled channels proteins from the two replicates normalized to median. **D.** Similar as in C. but for R-labelled channels. **E&F.** Global distribution of calculated RES for the K-labeled channel (E) and R-labeled (F). It can be observed that the two distributions are comparable with a similar dynamic range. **G.** No correlation (Pearson correlation ~0.02) was observed between the scores of proteins from R-labeled channels and K-labeled channels. **H.** LAMC1 estimated half-life from pSILAC data (~6 hours). **I.** The half-life of ST-TYR is ~1.5 h, close to previous reports.

SUPPLEMENTAL MATERIAL:

pSILAC data analysis

For pSILAC data analysis we have used the tetracycline EDEM2–inducible cell line (A375-ST-TYR-E2i), with the aim of obtaining differential kinetic profiles following EDEM2 expression. As observed in Figure S3B, EDEM2 expression is time-dependent (Figure S3B, middle panel), which also implies a time-dependent effect on the relative level of the ST-TYR (Figure S3B, upper panel) and possible on the potential substrates. Thus, we selected higher chase times (> 3h), which are considerably larger compared with the expected half-life of misfolded polypeptides. As an example, the ST- TYR has an estimated half-life of about 1.5 h [21]. Since EDEM2 is induced in only one of the samples, the evolution of tetracycline treated/control cells ratios E2i/C encoded by H/M or M/H ratios, depending on the replicate, in time, will reflect the differential regulation of proteins from the start of the experiment. Therefore, we define here the evolution score (ES) and the Relative Evolution Score (RES) which were used to score the kinetic profiles of proteins following EDEM2 induction. RES incorporates the Pearson correlation coefficient (*r*) and the non-negative maximum fold change relative value observed in the kinetic profile of the protein. We choose to incorporate the relative fold change amplitude in the scoring system in order to penalize proteins which show decreasing or increasing E2i/C ratios in time but with modest changes, so we sorted the identified proteins based on the calculated RES values. However, a particular consequence of the pSILAC workflow is that K and R channels, encode a different pool of molecules that allow the assessment of differences in both the degradation and synthesis rate between the two samples. The R, H and M channels, encode the pool of pre-existing molecules, before tetracycline treatment, while the K, H and M channels, encode all the proteins synthesized and degraded after the addition of tetracycline [43]. For this reason, RES values were calculated separately for proteins identified from K-derived peptides and proteins identified from R-derived peptides. Thus, this workflow allows the assessment of the differential expression of both, the old pool of molecules (before tetracycline treatment) and the new pool of proteins (after tetracycline treatment), considering also the half-life of proteins under study. Consequently, the fraction of protein molecules with short half-life affected by EDEM2 expression (such as those potential misfolded substrates) can be assessed by the E2i/C ratios using the K peptides and the protein molecules with longer half-life (such as the mature folded polypeptides) can be evaluated using the ratios from R peptides. This is best exemplified by the RES values of BiP (HSPA5) and HEXB obtained from R peptides (RES R) which are close to 0 (Figure S5B),

unlike the values from K peptides (RES K) that differ for the two proteins: BiP is co- upregulated with EDEM2 (RES K: +0.58) and HEXB is down-regulated (RES K: -0.56). Thus, the new pool of BiP and HEXB molecules is differentially impacted by the induction of EDEM2 expression, while the old pool of molecules remains largely unaffected. Analysis of the distribution for E2i/C ratios for each time-point, for both replicates on the two SILAC channels revealed a slightly larger distribution of K E2i/C ratios compared with the R E2i/C ratios (Figure S5C and D). This suggests that EDEM2 expression had a larger effect on the newly synthesized fraction of proteins (after tetracycline treatment), than on those of pre-existing molecules. Interestingly, the distribution of the K-derived ratios appeared to slightly narrow with increasing time, compared with the fairly constant E2i/C ratio distribution found on the R channel, suggesting a possible adaptation of cells to EDEM2 expression. We also compared the RES R and RES K distributions and found no major differences between the two scores. Both of the calculated RES values had a normal distribution and similar minimum values (Figure S5E and F) and found no correlation between them (Figure S5G). Finally, we also compared the absolute half-life for the LAMC1 and found an estimated half-life of around 6 h, close to the reported values of 5.5 h [98]. Similarly, our data confirmed the half-life of ST-TYR, close to 1.5 h (Figure S5I), as previously reported [21]. However, it should be considered that we did not aim to calculate absolute half-life values in the pSILAC workflow, as removing the cytosolic fraction by digitonin permeabilization could influence the absolute abundance values for some proteins.
